# Structural Studies Reveal the Role of Helix 68 in the Elongation Step of Protein Biosynthesis

**DOI:** 10.1128/mbio.00306-22

**Published:** 2022-03-29

**Authors:** Giuseppe Cimicata, Gil Fridkin, Tanaya Bose, Zohar Eyal, Yehuda Halfon, Elinor Breiner-Goldstein, Tara Fox, Ella Zimmerman, Anat Bashan, Natalia de Val, Alexander Wlodawer, Ada Yonath

**Affiliations:** a Department of Chemical and Structural Biology, The Weizmann Institute of Science, Rehovot, Israel; b Department of Organic Chemistry, Israel Institute for Biological Research, Ness Ziona, Israel; c Center for Molecular Microscopy, Center for Cancer Research, National Cancer Institute, National Institutes of Health, Frederick, Maryland, USA; d Center for Structural Biology, Center for Cancer Research, National Cancer Institute, Frederick, Maryland, USA; New York University School of Medicine

**Keywords:** ribosome, H68, translation, protein synthesis, PNA, antisense, cryo-EM, elongation

## Abstract

The ribosome, a multicomponent assembly consisting of RNA and proteins, is a pivotal macromolecular machine that translates the genetic code into proteins. The large ribosomal subunit rRNA helix 68 (H68) is a key element in the protein synthesis process, as it coordinates the coupled movements of the actors involved in translocation, including the tRNAs and L1 stalk. Examination of cryo-electron microscopy (cryo-EM) structures of ribosomes incubated for various time durations at physiological temperatures led to the identification of functionally relevant H68 movements. These movements assist the transition of the L1 stalk between its open and closed states. H68 spatial flexibility and its significance to the protein synthesis process were confirmed through its effective targeting with antisense PNA oligomers. Our results suggest that H68 is actively involved in ribosome movements that are central to the elongation process.

## INTRODUCTION

**FIG 1:**
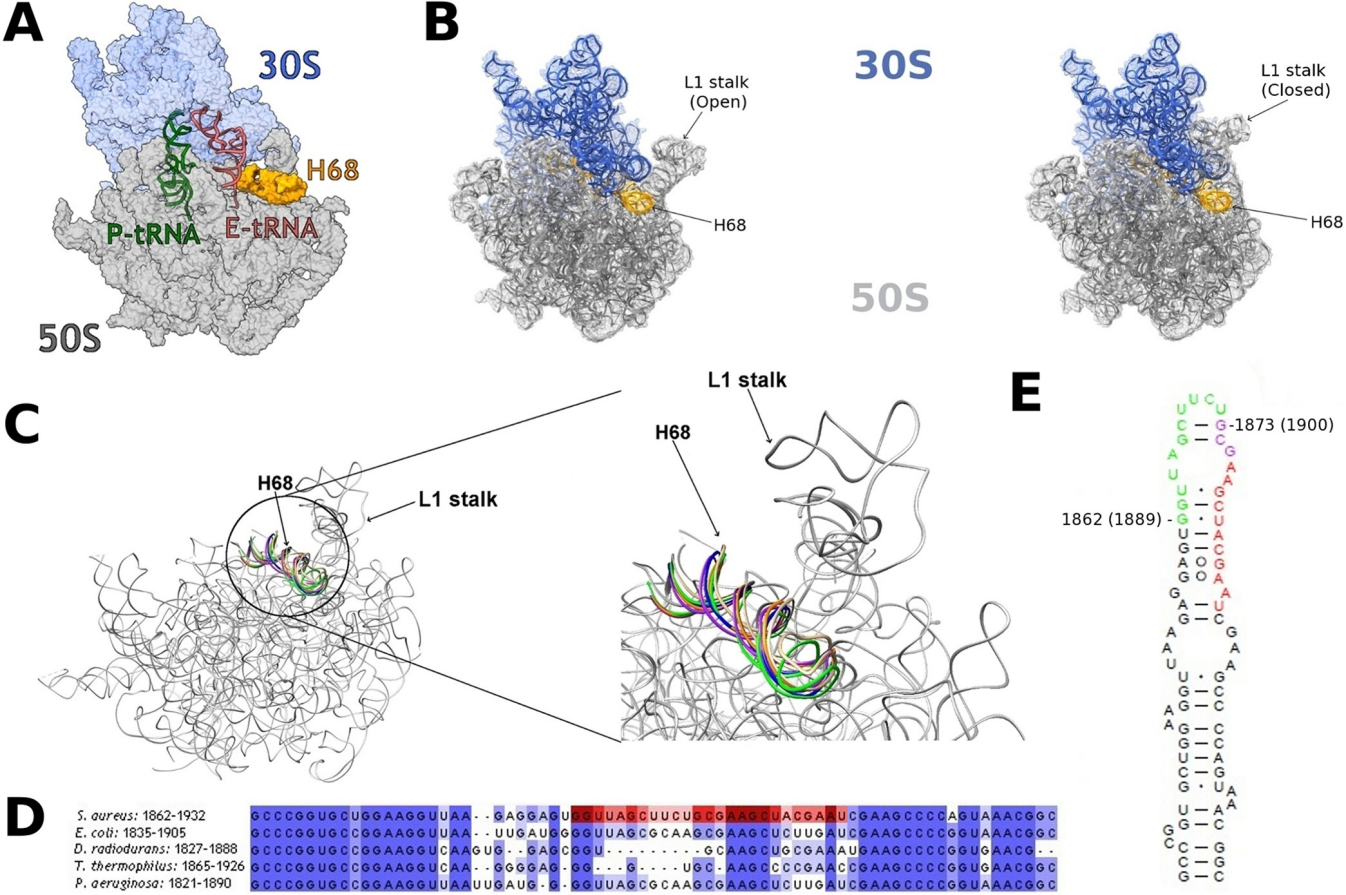
Location and orientation of rRNA H68 in bacterial ribosomes. (A) The structure of S. aureus ribosome complex (SA70S) highlighting the locations of the rRNA H68 (orange) at the surface of the 50S (gray) subunit. The 30S subunit, P-tRNA, and E-tRNA are shown in light blue, green, and red, respectively. (B) The L1 stalk in opened (PDB ID 4V9H) and closed (PDB ID 4V9D) conformations. The 50S and the 30S are represented in gray and blue, respectively. (C) Overlay of H68 in various structures of bacterial ribosome. S. aureus 50S (SA50S) is shown in gray (PDB ID 6HMA); E. coli (PDB ID 4V9H), T. thermophilus (PDB ID 6CFK), D. radiodurans (PDB ID 4WFN), and P. aeruginosa (PDB ID 6SPF) are shown in tan, purple, orange, blue, and green, respectively. (D) Sequence alignment of H68 in various bacterial organisms. The regions targeted with antisense oligomers are marked in red. The image was created using Jalview (47). (E) Two-dimensional structure of S. aureus H68. The regions targeted with antisense oligomers are marked in green (1862 series) and red (1873 series) with the number of the first residue of each series according to E. coli numbering (S. aureus numbering is shown in parenthesis). The nucleotides targeted by both series are marked in purple.

The ribosome, a macromolecular complex assembly consisting of ribosomal RNA (rRNA) and ribosomal proteins (rProteins), translates genetic information efficiently and accurately into proteins in all living cells. It is composed of large and small subunits, known in prokaryotes as 50S and 30S, respectively, based on their sedimentation coefficients (Fig. 1A). Apart from the ribosome, many other key players, including mRNA, tRNAs, and initiation, elongation, and termination protein cofactors, orchestrate the processes of protein synthesis.

Intricate functioning of the ribosome during protein biosynthesis is enabled by the highly integrated flexibility of some of its components (1). The movement of the structural element, called the L1 stalk, is a known example of such a large conformational change (2). This stalk belongs to a domain of the 50S subunit, and it is composed of one rProtein, called uL1, and three rRNA helices, H76 to H78. During translocation, the L1 stalk shifts from its so-called open state to its closed state (Fig. 1B) to assist the release of the E-site tRNA from the ribosome. These L1 movements are coordinated by rRNA H68 (3), (Fig. 1C), whose sequence near its base is highly conserved in bacteria (Fig. 1D). H68 is also a component of the B2b and B7a intersubunit bridges, which mediate interactions of the small and large ribosomal subunits with the incoming tRNA (4). Accordingly, H68 was shown to take part in the conformational changes underlying the ratchet-like motion of the 30S subunit (5). However, as all reported structures of the ribosome place H68 in the same position, this helix has been thus far considered a static actor in the dynamic scenario.

It is well-known that disparity of *in vitro* conditions, such as concentration of ions, may lead to the ribosome’s structural variability (6, 7). Specifically, it has been shown that increasing Mg^2+^ ion concentration from low values, i.e., 3.5 mM, to cytosolic ones, i.e., 10 mM, can dramatically alter the dynamic equilibrium between the classical and hybrid ribosomal states after peptidyl transfer in pretranslocation complexes (8). Fluorescence resonance energy transfer (FRET) experiments showed that differences in temperatures can also lead to different rRNA conformations, with kinetically stable complexes being favored at low temperatures (9). Moreover, it was extensively shown (10–13) that some cryocooling processes, which are routinely used in X-ray crystallography and cryo-EM to mitigate radiation damage, may hide high-energy conformations that are actually essential for protein function (14) and would be accessible at physiological temperatures. Accordingly, if a state is stabilized during purification of ribosomes, which is carried out at 4°C to prevent degradation, it would be impossible for them to acquire enough kinetic energy for relevant conformational changes since storing and structure determination operations are performed at cryogenic temperatures. We hypothesized that ribosome incubation at a physiologically relevant temperature, i.e., 37°C, prior to their imaging would allow the identification of functionally relevant conformational changes. Aiming to identify such rearrangements and following our hypothesis, we have conducted a thorough cryo-electron microscopy (cryo-EM) structural study of Staphylococcus aureus ribosomes that were incubated for various time durations (i.e., 0, 30, and 50 min) at 37°C.

As, indeed, such studies led to identification of H68 motions, we aimed to prove their significance for protein biosynthesis by targeting the helix with antisense-based peptide nucleic acid (PNA) oligomers. The efficacy of various antisense oligomer sequences to inhibit protein biosynthesis by binding to bacterial rRNA has been already proven by their effective targeting of clinically used antibiotic binding sites (15). Similarly, known functional sites on the small ribosomal subunit, such as the Shine-Dalgarno sequence (16), as well as on the large ribosomal subunit, like the α-sarcin loop (17) and H69 (18), were also successfully targeted by antisense oligomers. Accordingly, sets of specific anti-H68 PNA-based oligomers were designed, prepared, and examined for their ability to inhibit protein synthesis *in vitro*. Here, we present our structural results, highlighting the conformational variability of H68, as well as our *in vitro* studies toward its targeting.

## RESULTS

### Cryo-EM structural studies of S. aureus ribosomes.

**FIG 2:**
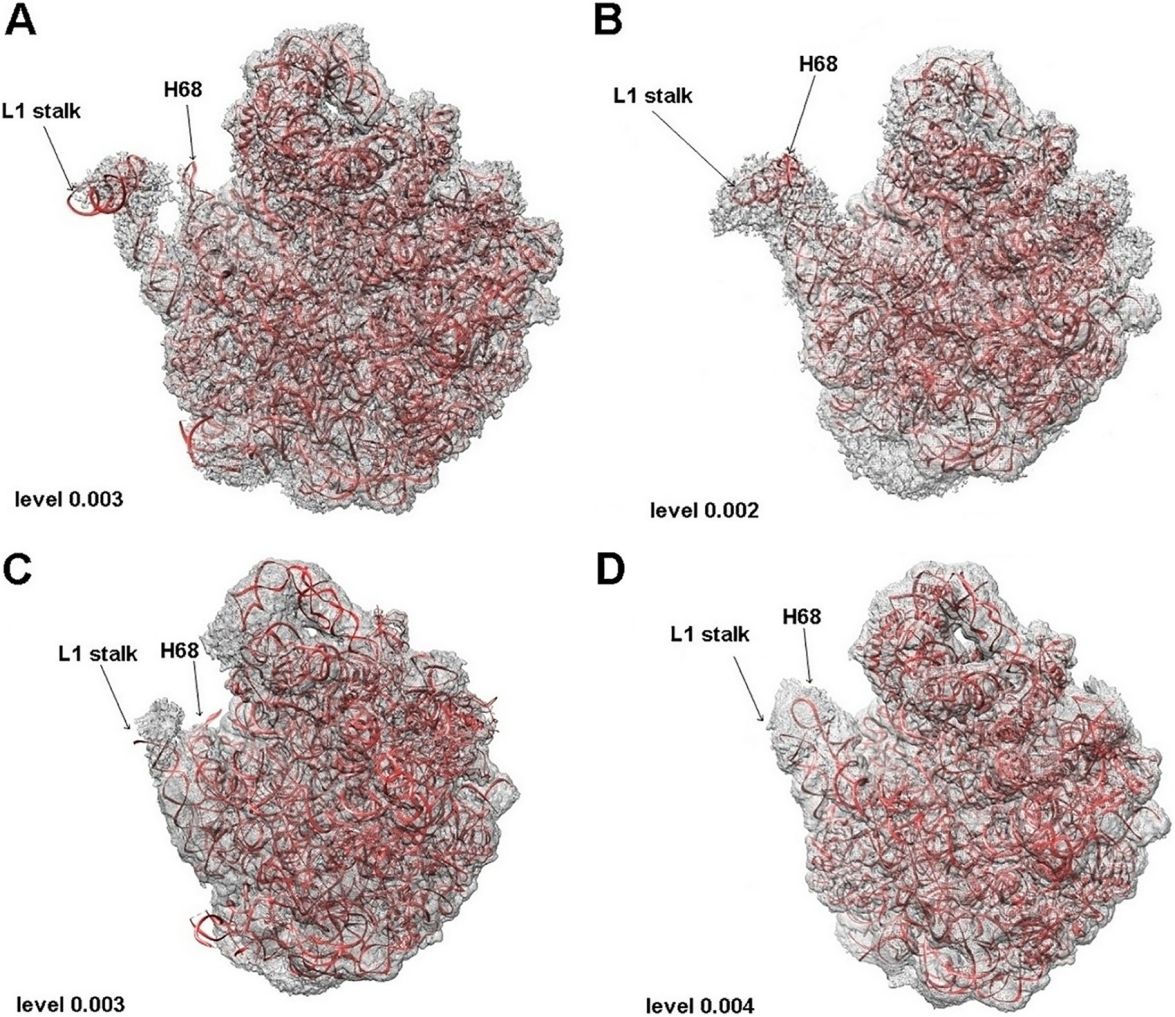
**(**A) SA50S_30 in its density. (B) SA50S_50 in its density. (C) Large ribosomal subunit from SA70S_30 in its density. (D) Large ribosomal subunit from SA70S_50 in its density. Contour level for each density is indicated on the bottom left corner.

Single-particle cryo-EM techniques were applied to identify ribosome flexible regions at 37°C. For that purpose, structures of the S. aureus large ribosomal subunit (SA50S) and of the whole ribosome (SA70S) were determined after incubation at physiological temperature for various time durations (Table 1). The nominal resolutions of the SA50S subunit structures were 2.65, 2.48, and 2.73 Å for 0, 30, and 50 min incubation time, respectively (SA50S_0, SA50S_30, and SA50S_50), whereas those of the SA70S ribosomes were 3.11 and 2.86 Å for 30 and 50 min, respectively (SA70S_30, SA70S_50) (Table S1, and Fig. S1A to E in the supplemental material). The previously published SA70S structure (19) (PDB ID 5TCU) was used as a baseline reference (no incubation) for comparison with the SA70S ribosome structures determined after incubation for 30 and 50 min. Also, other regions, e.g., rRNA helix 58, appear to undergo similar displacements (Fig. S2). However, since the peripheries of these structures were less well resolved, as is often the case with cryo-EM structures of the ribosome and other large complexes (Fig. S1A to E), they were not further analyzed here, as the resolution was considered too low to allow an unbiased modeling. Nevertheless, H68 was clearly visible; thus, this helix could be modeled in all five EM maps (Fig. 2 and Fig. S3).

**TABLE 1:** Behavior of rRNA H68 as observed in structures of SA50S and SA70S incubated at 37°C for different time durations

Ribosome subunit	Incubation time (min)	H68 behavior	PDB ID	Structure name
SA50S	0	No movement	6HMA	SA50S_0
SA50S	30	No movement	7ASM	SA50S_30
SA50S	50	Movement	7ASN	SA50S_50
SA70S (ancestor complex)	0	No movement	5TCU	SA70S_0
SA70S	30	No movement	7ASO	SA70S_30
SA70S	50	Movement	7ASP	SA70S_50

10.1128/mbio.00306-22.1 TABLE S1 Cryo-EM data collection and model refinement (for all new structures). Download Table S1, PDF file, 0.4 MB. Copyright © 2022 Cimicata et al. 2022 Cimicata et al. https://creativecommons.org/licenses/by/4.0/ This content is distributed under the terms of the Creative Commons Attribution 4.0 International license.

10.1128/mbio.00306-22.2 FIG S1 (Ai) Cryo-EM image processing flowchart for SA50S with no incubation. (Aii) Surface (left) and cross-section (right) rendering of the cryo-EM density maps colored according to local resolution. (Aiii) “Gold-standard” Fourier shell correlation (FSC) curves for the final three-dimensional map (black), unmasked map (green), masked map (blue), and phase-randomized masked map (red). (Bi) Cryo-EM image processing flowchart for SA50S with 30 minutes incubation. (Bii) Surface (left) and cross-section (right) rendering of the cryo-EM density maps colored according to local resolution. (Biii) Gold-standard FSC curves for the final three-dimensional map (black), unmasked map (green), masked map (blue), and phase-randomized masked map (red). (Ci) Cryo-EM image processing flowchart for SA50S with 50 minutes incubation. (Cii) Surface (left) and cross-section (right) rendering of the cryo-EM density maps colored according to local resolution. (Ciii) Gold-standard FSC curves for the final three-dimensional map (black), unmasked map (green), masked map (blue), and phase-randomized masked map (red). (Di) Cryo-EM image processing flowchart for SA70S with 30 minutes incubation. (Dii) Surface (left) and cross-section (right) rendering of the cryo-EM density maps colored according to local resolution. (Diii) Gold-standard FSC curves for the final three-dimensional map (black), unmasked map (green), masked map (blue), and phase-randomized masked map (red). (Ei) Cryo-EM image processing flowchart for SA70S with 50 minutes incubation. (Eii) cross-section (right) rendering of the cryo-EM density maps colored according to local resolution. (Eiii) Gold-standard FSC curves for the final three-dimensional map (black), unmasked map (green), masked map (blue), and phase-randomized masked map (red). Download FIG S1, PDF file, 16.8 MB. Copyright © 2022 Cimicata et al. 2022 Cimicata et al. https://creativecommons.org/licenses/by/4.0/ This content is distributed under the terms of the Creative Commons Attribution 4.0 International license.

10.1128/mbio.00306-22.3 FIG S2 Overlay of SA50S_0 (orange) and SA50S_50 (blue) in the SA50S_50 sharpened map (yellow) and unsharpened map (gray). The moveable H58 was not modeled in SA50S, as the resolution of the map was too low to allow an unbiased modeling. Download FIG S2, PDF file, 0.9 MB. Copyright © 2022 Cimicata et al. 2022 Cimicata et al. https://creativecommons.org/licenses/by/4.0/ This content is distributed under the terms of the Creative Commons Attribution 4.0 International license.

10.1128/mbio.00306-22.4 FIG S3 H68 in the EM density maps and the respective fitted structures shown in a vertical images of front and top views. (A) SA50S_30; (B) SA50S_50; (C) SA70S_30; (D) SA70S_0. Download FIG S3, PDF file, 0.4 MB. Copyright © 2022 Cimicata et al. 2022 Cimicata et al. https://creativecommons.org/licenses/by/4.0/ This content is distributed under the terms of the Creative Commons Attribution 4.0 International license.

Since the density of H68 was visible in all the EM maps corresponding to 50 min incubation time (Fig. 2B and D), we suggest that the displacement from the previously determined position indicates an intrinsic dynamic property of this region which is thus essential for ribosomal activity.

**FIG 3:**
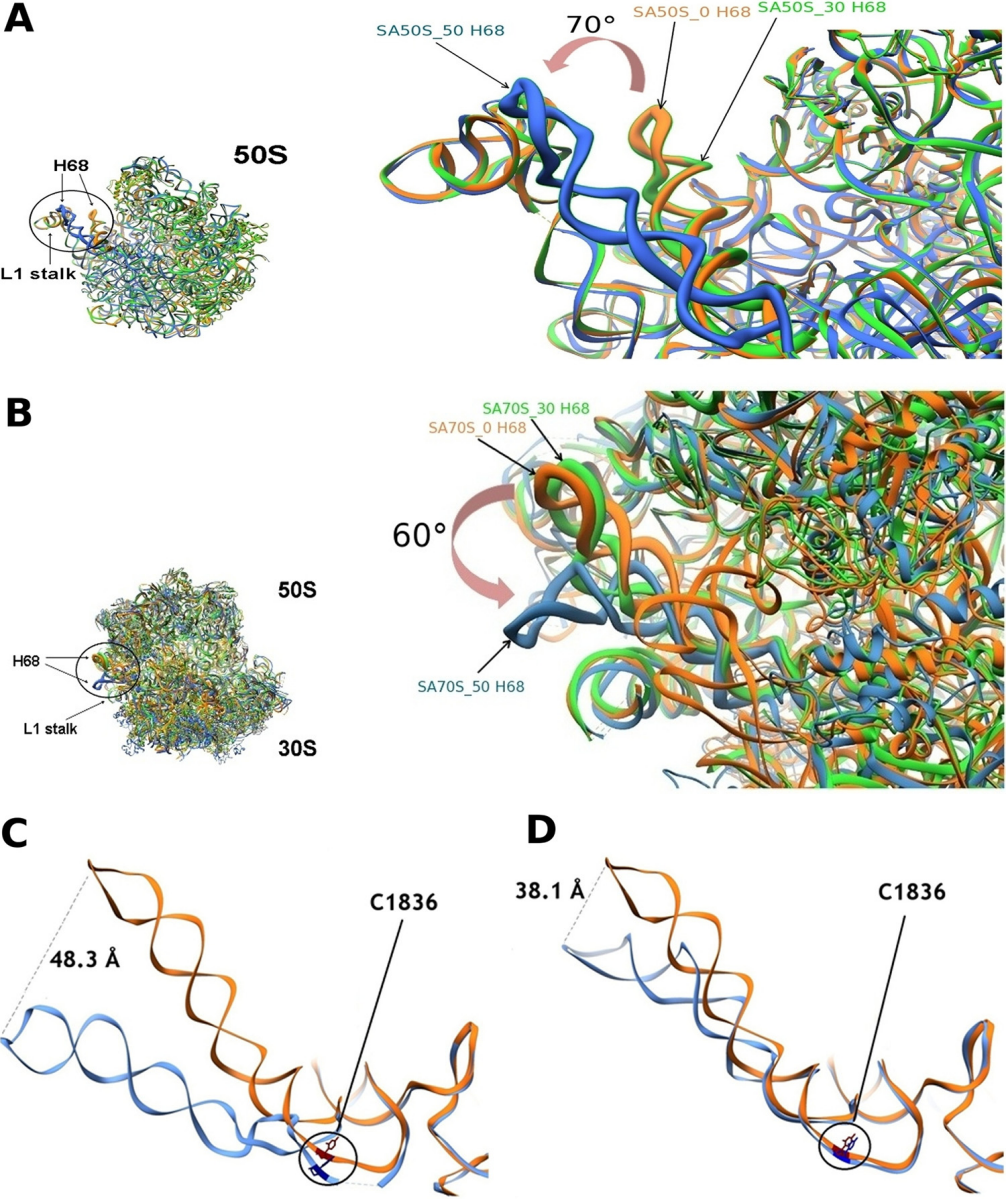
An overlay of ribosome structures before and after incubation. (A) Overlay of SA50S structures. SA50S_0 (0 min incubation), SA50S_30 (30 min incubation), and SA50S_50 (50 min incubation) are shown in orange, green, and blue, respectively. On the right, detail of the large ribosomal subunit showing the movement of H68 marked by an arrow. The angle between the moved and unmoved helix was calculated considering residue C1895 at the base of the helix as the point of intersection. (B) Overlay of SA70S structures. SA70S_0 (0 min incubation), SA70S_30 (30 min incubation), and SA70S_50 (50 min incubation) are shown in orange, green, and blue, respectively. On the right, detail of the ribosome showing the movement of H68 marked by an arrow. The angle between the moved and unmoved helix was calculated considering residue C1895 at the base of the helix as the point of intersection. (C) Ribbon representations of H68 from the SA50S structure obtained without incubation (orange) and H68 from the SA50S structure obtained after 50 min incubation (cornflower blue). The rRNA nucleotides C1836 in each of the above-mentioned helices are shown in red and navy, respectively. The distance between H68 in SA50S_0 versus SA50S_50 structures is shown by a dashed line. (D) SA70S structure obtained without incubation (orange) and SA70S after 50 min incubation (cornflower blue); position C1836 is highlighted in red and navy, respectively. The distance between H68 in SA70S_0 versus SA70S_50 structures is shown by a dashed line.

Superposition of both 50S and 70S ribosome structures acquired after 50 min incubation time on the ribosome structures obtained directly without incubation and after 30 min incubation indicated a shift of H68 toward the L1 stalk (Fig. 3A and B). Dislocation of ∼50 Å with an angle of rotation of ∼70° and ∼40 Å with an angle of rotation of ∼60° in respect to the previously known helix position were observed between the tips of H68 in the 50S and 70S, respectively (Fig. 3C and D). This movement appears particularly significant considering that H69, well-known for its flexibility (20), shows a displacement of only ∼15 Å both in the SA50S_50 and the SA70S_50 (Fig. S4). This H69 movement in opposite directions in SA50S_50 versus SA70S_50 structures suggests it is due to the presence or absence of the small ribosomal subunit rather than to the movement of the nearby H68.

10.1128/mbio.00306-22.5 FIG S4 (A) Overlay of SA50S_0 (orange) and SA50S_50 (blue) H69. (B) Overlay of SA70S_0 (orange) and SA70S_50 (blue) H69. Download FIG S4, PDF file, 0.2 MB. Copyright © 2022 Cimicata et al. 2022 Cimicata et al. https://creativecommons.org/licenses/by/4.0/ This content is distributed under the terms of the Creative Commons Attribution 4.0 International license.

The restricted displacement of H68 observed in the 70S ribosome is also probably due to the presence of the 30S subunit, which seems to reduce the flexibility of this helix. The movement of the rRNA residue C1836 (Escherichia coli numbering used throughout), which is a part of the intersubunit bridge B2b observed in the SA50S_50 structure but barely seen in the SA70S_50 structure (Fig. 3D), further supports the assumed differential effect of the interactions between 50S and 30S on H68 flexibility. The heterogeneous three-dimensional refinement of the particles used for the three-dimensional reconstruction revealed a differential effect of the small ribosomal subunit on the dynamics of the rearrangement of H68. In fact, while for SA70S_30, the heterogeneous three-dimensional refinement results in two identical classes with H68 in the original position, two distinct classes can be described for SA50S_30. One class accounts for 79% (144,826 particles) of the particles used for the three-dimensional refinement with H68 in the original position, and the other one accounts for 21% (38,658 particles) of the particles, with H68 already showing the rearranged conformation (Fig. S5). After 50 min incubation, both 50S and 70S show two classes with rearranged conformation of H68. These data suggest that the rearrangement happens faster when the small ribosomal subunit is missing.

10.1128/mbio.00306-22.6 FIG S5 (A) The class with H68 in the original position obtained from the heterogeneous three-dimensional refinement of the particles used for the three-dimensional reconstruction of SA50_30 is shown in blue. The rRNA from SA50S_0 (PDB ID 6HMA) is shown in mulberry red. (B) The class with the rearranged H68 obtained from the heterogeneous three-dimensional refinement of the particles used for the three-dimensional reconstruction of SA50_30 is shown in yellow. The rRNA from SA50S_50 (PDB ID 7ASN) is shown in mulberry red. Download FIG S5, PDF file, 0.4 MB. Copyright © 2022 Cimicata et al. 2022 Cimicata et al. https://creativecommons.org/licenses/by/4.0/ This content is distributed under the terms of the Creative Commons Attribution 4.0 International license.

### Design of the antisense PNA oligomers.

The identification of H68 flexibility prompted us to hypothesize that this helix plays a central role in the ribosome’s mechanism of action. In order to examine this notion, two sets of PNA oligomers (Fig. 1E, and Fig. S6) were designed based on sequence complementarity with H68 and tested for their capability to inhibit protein synthesis *in vitro*. In particular, we aimed at targeting the region of H68 that becomes, upon its motion, adjacent to the L1 stalk. This H68 L1 stalk-facing region was divided into two target sites, namely, nucleotides 1862 to 1875 and 1873 to 1885. A set of oligomers with various lengths, i.e., 10, 12, and 14 bases, was designed and prepared for each of the selected H68 target sites, as it was predicted that the length of oligomers might affect their binding propensity and inhibition activity (21).

10.1128/mbio.00306-22.7 FIG S6 Chemistry of PNA molecule. Download FIG S6, PDF file, 0.02 MB. Copyright © 2022 Cimicata et al. 2022 Cimicata et al. https://creativecommons.org/licenses/by/4.0/ This content is distributed under the terms of the Creative Commons Attribution 4.0 International license.

### *In vitro* inhibition of protein synthesis.

Following preparation and purification of the PNA oligomers, their capacity to inhibit protein synthesis was tested using an *in vitro* cell-free transcription/translation assay based on luciferase as the reporter gene (Fig. S7A to F). As seen in Table 2, targeting both sites led to effective inhibition of protein synthesis with 50% inhibitory concentration (IC_50_) values in the low micromolar range. Remarkably, the slope calculated for all the oligomers is lower than 1, suggesting that only one oligomer binds to each ribosome. In addition, our results show that 12-mer PNA oligomers have a higher inhibition activity than the shorter 10-mer, while transition to the longer 14-mer resulted only in a minor increase of inhibitory activity. The 12-mer oligomers, which appeared optimal in respect to effective inhibition and future penetration through the bacterial cell wall, were therefore selected for the following binding experiments.

**TABLE 2:** The sequences of PNA oligomers and their *in vitro* cell-free transcription/translation inhibition activity measured using S. aureus ribosomes^*a*^

PNA oligomers according to E. coli numbering^*b*^	Sequence (5′→3′)	IC_50_ (μM)	Slope
1862-10 (1889-10)	GAAGCTAACC	20.6 ± 8.7	0.3 ± 0.03
1862-12 (1889-12)	CAGAAGCTAACC	2.2 ± 0.9	0.5 ± 0.07
1862-14 (1889-14)	CGCAGAAGCTAACC	1.3 ± 0.7	0.5 ± 0.09
1873-10 (1900-10)	GTAGCTTCGC	6.3 ± 2.4	0.6 ± 0.10
1873-12 (1900-12)	TCGTAGCTTCGC	4.9 ± 1.2	0.7 ± 0.06
1873-14 (1900-14)	ATTCGTAGCTTCGC	3.8 ± 0.5	0.7 ± 0.06

a Errors reported are standard errors of duplicate experiments.

b S. aureus numbering is given in parentheses.

10.1128/mbio.00306-22.8 FIG S7 (A) Inhibition assay using PNA 1862-10 and S. aureus ribosome. Fitting of the luminescence data for oligonucleotide was performed with GraFit. (B) Inhibition assay using PNA 1862-12 and S. aureus ribosome. Fitting of the luminescence data for oligonucleotides was performed with GraFit. (C) Inhibition assay using PNA 1862-14 and S. aureus ribosome. Fitting of the luminescence data for oligonucleotides was performed with GraFit. (D) Inhibition assay using PNA 1873-10 and S. aureus ribosome. Fitting of the luminescence data for oligonucleotide was performed with GraFit. (E) Inhibition assay using PNA 1873-12 and S. aureus ribosome. Fitting of the luminescence data for oligonucleotide was performed with GraFit. (F) Inhibition assay using PNA 1873-14 and S. aureus ribosome. Fitting of the luminescence data for oligonucleotide was performed with GraFit. (G) Relative inhibition of mismatch sequences compared to the matching sequences. (Gi) PNA oligonucleotides 1862 series (1889 in S. aureus numbering). (Gii) PNA oligonucleotide 1873 series (1900 in S. aureus numbering). Download FIG S7, PDF file, 0.3 MB. Copyright © 2022 Cimicata et al. 2022 Cimicata et al. https://creativecommons.org/licenses/by/4.0/ This content is distributed under the terms of the Creative Commons Attribution 4.0 International license.

### Binding of PNA oligomers to the SA50S.

**FIG 4:**
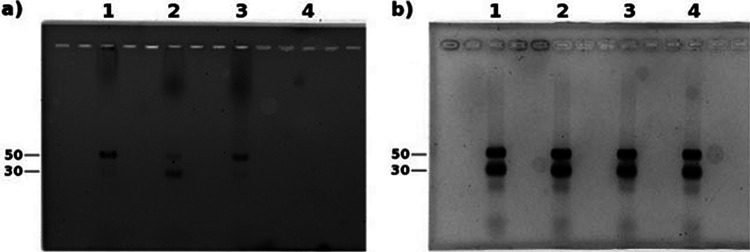
Binding assay of PNA oligomers to the S. aureus ribosomal subunits using native agarose gel. Shown are 12-mer PNA oligomers and the Shine-Dalgarno with S. aureus small and large subunits Lane 1- 1862-12, Lane 2- 1862-12-2, Lane 3-1873-12. Lane 4-negative control, no oligomer used. The gel was read at 494 nm (a) and, after ethidium bromide addition, at 600 nm (b).

To evaluate the binding affinity between the various PNA oligonucleotides and SA50S, the fluorescein-labeled 12-mer oligomer of both sets was incubated with SA50S and SA30S, and the mixtures were run on a nondenaturing agarose gel. A mismatched sequence of the oligomer 1862-12 holding 2 mismatches, 1862-12-2, was used as a control. The intense bands in the gel (read at 494 nm) (Fig. 4) indicate that, indeed, the oligonucleotides bind selectively to the SA50S, whereas the control shows weak binding to the SA50S and appears to bind the SA30S more tightly. Importantly, based on the intensity of the bands, it appears that the most active PNA inhibitor (i.e., 1862-12) also exhibited the strongest binding.

To further investigate the specificity of interactions between the antisense oligomers and their designed targets, the effect of introduction of mismatches to the sequences on their activity was also assessed. Accordingly, the inhibition activity of oligomers holding 2 or 3 mismatches of each sequence was evaluated using the same *in vitro* transcription/translation assay (Table 3).

**TABLE 3:** Sequences of mismatched PNA oligonucleotides^*a*^

PNA oligomers according to E. coli numbering^*b*^	Sequence (5′→3′)
1862-12-2 (1889-12-2)	CAATAGCTAACC
1862-12-3 (1889-12-3)	CAGTTCCTAACC
1873-12-2) (1900-12-2)	TCAGAGCTTCGC
1873-12-3 (1900-12-3)	TAAGAGCTTCGC

a Mismatches are underlined.

b S. aureus numbering is given in parentheses.

Indeed, these mismatched sequences led to poor inhibition activity, which prevented reliable estimation of their IC_50_ values. Nonetheless, a comparison of their activity at high concentrations to that of the fully complementary oligomers at the same concentrations clearly shows that the former exhibit much lower activity (Fig. S7G). This pattern suggests there is a direct dependence between the oligomer sequences and their activity, and accordingly, it implies that they are most likely indeed interacting with their designed target sites on H68.

## DISCUSSION

**FIG 5:**
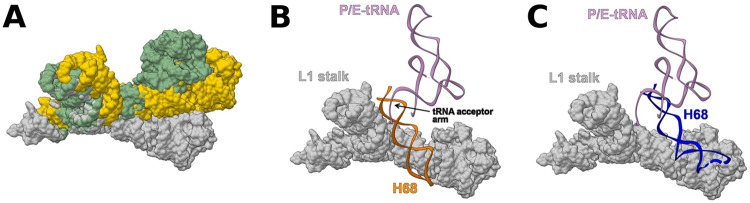
Relative conformations of L1 stalk and H68. (A) L1 stalk in open (21) (PDB ID 4V9D), intermediate (19) (PDB ID 5TCU), and closed (22) (PDB ID 4V9H) conformations is shown in gray, gold, and green, respectively. (B) L1 stalk in open conformation (22) (PDB ID 4V9H) overlaid with H68 from SA70S_0 (orange) and P/E-tRNA (19) (pink) (PDB ID 5TCU) is shown in gray. (C) L1 stalk in open conformation (22) (PDB ID 4V9H) overlaid with P/E-tRNA (19) (pink) (PDB ID 5TCU) and H68 from SA70S_50S (blue) is shown in gray.

**FIG 6:**
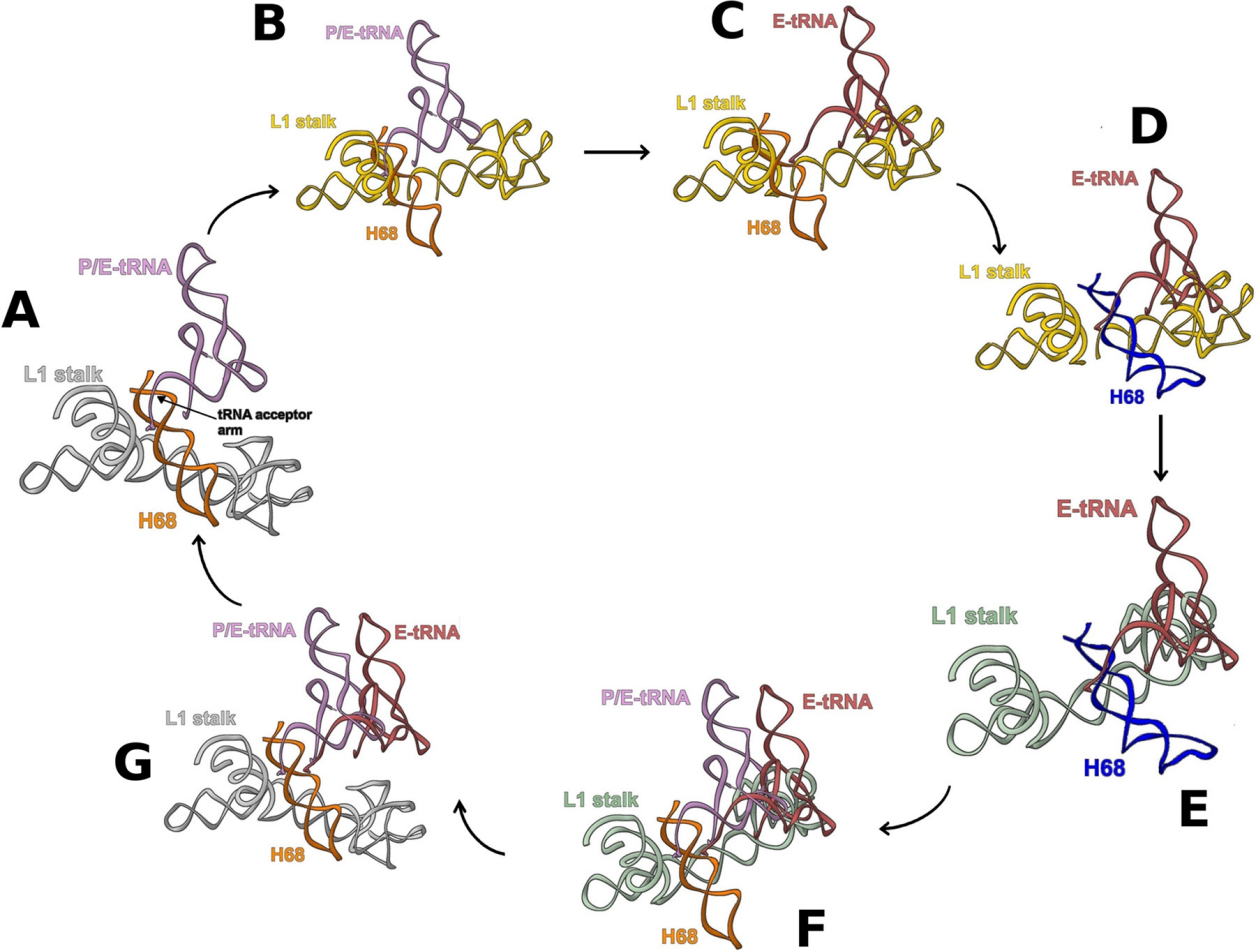
Concerted movement of tRNA, L1 stalk, and H68. (A) L1 stalk in open conformation (from E. coli 70S structure (21) (PDB ID 4V9D) overlaid on P/E-tRNA (22) (PDB ID 4V9H) and H68 from SA70S_0 (19) (PDB ID 5TCU) are shown in gray, pink, and orange, respectively. (B) The L1 stalk, shown in gold, moves to the intermediate conformation (19) (PDB ID 5TCU). (C) The tRNA, shown in red, moves to the E-site (19) (PDB ID 5TCU). (D) H68, in blue, moves to its new conformation. Residue U2098 is shown in light green with atoms and bonds represented. (E) The movement of H68 pushes the L1 stalk, shown in green, to the closed conformation. (F) The arrival of a new hybrid P/E-tRNA, shown in pink, pulls H68, shown in orange, to its original position. (G) The L1 stalk, shown in gray, has now room to return to the open conformation, and the E-tRNA, shown in red, can leave the ribosome.

This study aimed at the identification of conformational changes in the ribosome at functional temperature (37°C) to provide further insight into the ribosome mechanism of action and for future development of antisense-based antibiotics. Following our findings of H68 movements upon its incubation for 50 min at 37°C, we analyzed H68 interactions with the L1 stalk and tRNA to specifically shed light on its role during translocation. Thus, we superimposed H68 from SA70S_0 and SA70S_50 structures on the L1 stalk both in open (22) (PDB ID 49VD) and closed (23) (PDB ID 4V9H) conformations, as well as on a third conformation, namely, the intermediate one, positioned between the open and the closed states (19) (Fig. 5A), the tRNA in the E-site (19) (PDB ID 5TCU), and in the hybrid P/E state (23) (PDB ID 4V9H) (Fig. 6).

These overlays show that the 3′ end acceptor arm of the P/E tRNA is involved in a minor groove contact with the SA70S_0 H68 at positions 1850 to 1852 and 1892 to 1893 (Fig. 5B). However, upon incubation for 50 min at 37°C, H68 conformation changes (SA70S_50 structure) so that the minor groove interactions with the tRNA are lost (Fig. 5C).

We suggest that during translocation, the same (or a very similar) movement of the H68 might be triggered by the shift of the tRNA from the hybrid P/E configuration to the classical E-state, as the minor groove interactions are broken when the tRNA is in the exit site (Fig. 5C). Moreover, by proceeding to the E-site, the tRNA molecule stabilizes the L1 stalk in an intermediate position between the open and the closed state (Fig. 6C). This state, which is accessible by thermal fluctuations of the H76/H75/H79 junction (24), is stabilized by interactions between its elbow and the head domain of the L1 stalk (24, 25), as can be deduced by the finding that the L1 stalk is often detected in this position in ribosome structures determined by X-ray (26, 27) and by cryo-EM (19) with an E-site-bound tRNA. The transition of the L1 stalk from the open to the intermediate conformation leaves then sufficient space for H68 movement to its newly found conformation (Fig. 6D). In turn, such H68 movement might be responsible for the shift of the L1 stalk from the intermediate to the closed state (Fig. 6E). Since the L1 stalk intermediate state is better stabilized by E-tRNA, whereas the closed state is more stable with the tRNA in the hybrid P/E state (28), the H68 movement must intervene to destabilize the L1 stalk in the intermediate conformation when the E-site is occupied in order to force it to the closed one. In particular, H68 approaches the region of the L1 stalk starting with residue U2098, which was elucidated as a major inflection point (with U2092) of the stalk (3) (Fig. 6D), buttressing the hypothesis that H68 movement plays an active role in the L1 stalk conformational change. Computational studies showed that tRNA is released from the ribosome when the L1 stalk is at its open position, but it is unclear what triggers this motion (29). It may well be that, when a new tRNA reaches the P/E state, H68 moves to the original position to establish a minor groove interaction with the new P/E tRNA (Fig. 6F), leaving space for the L1 stalk to go backward (Fig. 6G) and then triggering the release of the deacylated tRNA in the E-site from the ribosome.

The necessity of a long 50 min incubation at 37°C to detect the H68 movement is not surprising considering that in large ribozymes, such as the ribosome, *in vitro* conformational changes usually require very long times (30). Moreover, this conformational rearrangement seems to be related to the translocation step, which is accelerated by elements like the elongation factor G (31) and an aminoacylated tRNA in the A-site (32), whose absence during the incubation might have contributed to slowing down the movement of the helix. The functional relevance of the movements of H68 to the protein synthesis process was demonstrated using PNA oligomers designed to bind selectively to those regions of H68 that are involved in the interactions with the L1 stalk. The specific interactions of these oligomers with H68 are shown by their selective binding to the large ribosomal subunit and by the reduced activity exhibited by the mismatched sequences. Such interactions lead to inhibition of protein synthesis with IC_50_ values at low micromolar range because of the interference with crucial interactions of H68 and limitation of its flexibility.

In conclusion, our findings suggest that the previously determined position of the rRNA H68 represents a single stable state, whereas changes in temperature can trigger a shift to an alternative conformation, which plays an important role in coordinating translocation.

## MATERIALS AND METHODS

### Bacterial growth.

S. aureus *strain* RN4220 was grown overnight at 37°C. Cells were harvested at optical density at 600 nm (OD_600_) of ∼1.5. The bacterial culture was centrifuged three times for 15 min each at 5,000 rpm using a GS-3 rotor at 4°C and the supernatant discarded. The pellet was resuspended and centrifuged at 4,000 rpm for 10 min at 4°C using a GS-3 rotor in a tabletop centrifuge. The supernatants were discarded, and the wet pellets (cells) were weighed. In order to disrupt the cell membranes, the samples were resuspended in 10 mM Tris-acetate buffer, pH 8.0, 14 mM Mg acetate, 1 M KCl, 1 mM dithiothreitol (DTT), and 50 μg/mL lysostaphin and were incubated at 37°C for 1 h and periodically inverted. The lysates were centrifuged for 30 min at 160,00 rpm at 4°C using an SS-34 rotor for removing cell debris. The supernatants were incubated in 670 mM Tris-acetate buffer, pH 8.0, 20 mM Mg acetate, 7 mM DTT, 7 mM Na_3_-phosphoenolpyruvate, 5.5 mM ATP, 70 mM from each amino acid, and 1.9 mg pyruvate kinase at 37°C for 30 min and dialyzed overnight at 4°C against 10 mM buffer of Tris-acetate, pH 8.0, 14 mM Mg acetate, 60 mM K-acetate, and 1 mM DTT dialysis buffer. The extract was then flash frozen and stored at −80°C.

### Ribosome purification.

Cell extract was layered on a 1.1 M sucrose cushion, H10M15N100K*15*βMe6, pH 8.0 (10 mM HEPES pH 8.0, 15 mM MgCl_2_, 100 mM NH4Cl, 50 mM KCl, and 6 mM β-mercaptoethanol), and ultracentrifugated twice, each time at 55,000 rpm using a Ti-70 rotor at 4°C for 17 h. The pellet was dissolved in H10M15N150βMe6, pH 8.0, at 4°C. Ribosomal subunits were then separated by zonal ultracentrifugation using a Ti-15 zonal rotor with 8 to 40% sucrose gradient at low Mg^2+^ (1 mM MgCl_2_) for 17.5 h at 27,000 rpm. After separation, the concentration of Mg^2+^ was adjusted to 10 mM, and the ribosomal subunits fractions were concentrated using sequential centrifugations. The samples were kept in H10M15N60K15 (10 mM HEPES pH 8.0, 15 mM MgCl_2_, 60 mM NH4Cl, 15 mM KCl), pH 7.6, brought to a final concentration not exceeding 1,000 *A*_260_ mL^−1^, and then flash frozen for storage at −80°C.

### PNA oligonucleotide synthesis.

The PNA oligomers were synthesized manually on Fmoc-Lys(Boc)-NovaSyn TGA resin (loading, 0.18 mmol/g; Merck). The synthesis followed typical PNA Fmoc protocols. In brief, deprotection of Fmoc from Lys as well as the PNA monomers was performed using a solution of 20% piperidine in dimethylformamide (DMF), and couplings were performed with 4 equivalents of Fmoc-PNA monomers (PolyOrg. Inc.), *N*,*N*,*N*′,*N*′-tetramethyl-*O*-(7-azabenzotriazol-1-yl)uronium hexafluorophosphate (HATU) and 1-hydroxybenzotriazole (HOBT; Chem-Impex International Inc.), and 8 eq of *N*,*N*-diisopropylethylamine (DIEA) (Sigma-Aldrich) in DMF. The reactions vessels (polypropylene solid-phase extraction [SPE] tubes, 3 and 6 mL; frits, lids, and stopcocks were purchased from Silicol) were typically shaken for 3 to 4 h, and then the resin was washed three times with DMF and twice with dichloromethane (DCM) (Macron). Success of couplings, as well as Fmoc removal, was monitored by ninhydrin. In all cases, only one cycle of coupling was required. Simultaneous deprotection of Bhoc and cleavage from the resin were performed using a 95:5 mixture of trifluoroacetic acid (TFA)/m-cresol. After ∼3 h at room temperature, the solution was transferred to cold ether to precipitate the product; this step was repeated three times. After decantation of ether, water was added to the product, and it was lyophilized. The products were then purified using high-pressure liquid chromatography (HPLC) (Waters Preparative HPLC; 2545 quaternary gradient module equipped with a Waters 2489 UV/visible detector using a gradient of 5% to 40% acetonitrile/0.1% TFA in water/0.1% TFA over 30 min), and their identity and purity were verified by liquid chromatography-mass spectrometry (LC-MS) (Waters). For labeling of the oligonucleotides PNA with 5′-6-carboxyfluorescein (Sigma-Aldrich), the Fmoc-aminoethylethanolamine (AEEA)-OH linker (PolyOrg. Inc.), was first coupled to the resin using the protocol described above. Following the linker Fmoc deprotection, a mixture of 5 eq 5′,6-carboxyfluorescein, 5 eq of HOBt, 5 eq of benzotriazol-1-1-hexafluorophosphate-oxy-Tris-pyrrolidine-phosphonium (PyBOP), and 10 eq of DIEA in DMF was added to the resin after 10 min of preactivation. The tube was then covered with aluminum foil and shaken overnight. A second coupling using the same amounts was performed for an additional ∼3 h. The cleavage from the resin was performed as described above.

### *In vitro* inhibition of translation.

The inhibition effect of the PNA oligonucleotides on S. aureus ribosomes was tested in a bacterial coupled transcription-translation assay system where the expression of the luciferase gene was measured (33). The luciferase gene was inserted into a plasmid with T7 RNA polymerase promoter. Serial dilutions of each oligonucleotide in water (the concentrations of the stock solutions were determined by measuring their absorbance at 260 nm) from 1 mM to 64 nM were prepared; a reaction mixture, including 0.12 (vol/vol) E. coli S100 lysate, 300 nM SA30S, 300 nM SA50S, 1.3 mM amino acids mix, 0.25 mg/mL creatine kinase, 0.027 mg/mL T7 RNA polymerase, 0.003 μg/mL luciferase plasmid, 174 μg/mL E. coli tRNA mixture, 160 mM HEPES KOH (pH 7.5), 6.5% polyethylene glycol 8000 (PEG 8000), 0.074 mg/mL tyrosine, 208 mM potassium glutamate, 1.8 mM DTT, 1.3 mM ATP, 0.86 mM GTP, 0.86 mM CTP and 0.86 mM UTP, 0.663 mM cyclic AMP (cAMP), 83 mM creatine phosphate, 0.036 mg/mL folinic acid, 28 mM ammonium acetate, and 14.8 mM magnesium acetate was prepared; 30 μL of the reaction mixture was incubated for 50 min at 37°C in the presence of different concentrations of the various oligonucleotides; the reactions were terminated with the addition of 48 μL of water. Luciferin assay reagent (LAR; Promega) at 5:3 (luciferase/reaction mix) volume ratio was added to the mixture, and photoluminescence was instantly measured; dose-response curves were fitted to the experimental data with GraFit (34) to calculate IC_50_ values using the 4-parameter equation

$$y=\frac{d-c}{1+{\left(\frac{x}{{\text{IC}}_{50}}\right)}^{s}}\;+\;c$$

where *x* is the concentration of inhibitor, *y* is the luminescence, *c* is the lower limit of the curve, *d* is the upper limit of the curve, and *s* is the slope of the curve.

### S. aureus agarose gel electrophoresis.

Samples were prepared by mixing 4 μL Milli-Q H_2_O with 2 μL of 2-μM ribosomal subunits (670 nM); 2 μL of 2 μM fluorescein-labeled oligonucleotide (200 nM) dissolved in Milli-Q H_2_O. We added 3 μL of RNasin to each sample. The sequence of the oligomer used as a positive control is CAGTTCCTAACC. Samples were loaded onto a 1.5% agarose gel and electrophoresed in 1×Tris-borate-EDTA (TBE) buffer. The gel was run for 120 min using 80 V at 4°C.

### Cryo-EM data collection and acquisition.

Upon incubation at 37°C for different intervals of time (0 min for SA50S_0, 30 min for SA50S_30 and SA70S_30, and 50 min for SA50S_50 and SA70S_50), 3 μL of a 1.0-mg/mL ribosome solution was applied on glow-discharged holey carbon grids (Quantifoil R2/2; 300 mesh). The grids were blotted for 3.0 s at 4°C and 100% humidity and vitrified in liquid ethane precooled by liquid nitrogen using a Vitrobot Mark IV (FEI). Cryo-EM micrographs were recorded at liquid nitrogen temperature on a Titan Krios electron microscope (FEI) operating at 300 kV. Micrographs of SA50S_0, SA50S_30, and SA50S_50 were recorded on a Gatan K2 Summit at a magnification of ×29,000; pixel size, 0.859 Å·px^−1^, defocus range of −0.7 to −1.3 μm, and total dose 40 e/Å^2^. These data sets were collected at the High-Resolution Imaging Section at the Center for Molecular Microscopy, Frederick National Laboratory for Cancer Research, Frederick, Maryland, USA. Micrographs of SA70S_30 and SA70S_50 were recorded on a Falcon 3 (FEI) at a magnification of ×127,000, pixel size, 1.1 Å·Å·px^−1^, defocus range −0.5 to −1.50 μm, and total dose of 40 e/Å^2^. These data sets were collected at the Weizmann Institute Electron Microscopy Unit, Israel.

### Cryo-EM image processing and three-dimensional reconstructions.

Cryo-EM images were subjected to motion correction by using MotionCor2 (35). Contrast transfer function parameters for each micrograph were determined by CTFFIND4 (36). Particle selection, as well as two-dimensional and three-dimensional classifications, was performed in RELION 3.0 (37, 38). The resulting particle projections were subjected to further refinement with alignment focusing on the small subunit (SSU) and large subunit (LSU). The particles used for the three-dimensional refinement were migrated to cryoSPARC (39) where a heterogeneous classification for 2 classes was performed. Reported resolutions are based on the gold-standard Fourier shell correlation using the 0.143 criteria (Fig. S1A to E). Local resolution was determined by using ResMap (40) with half-reconstructions as input maps (Fig. S1A to E).

### Model building and refinement.

Model building was performed by fitting the reported model of the S. aureus ribosome PDB ID 4WCE for SA50S and S. aureus PDB ID 5TCU for SA70S to the calculated density map using Chimera (41). The docked model was manually manipulated with Coot (42) real space and geometry-restraint commands to fit into the density maps. Model refinement was performed with a combination of PHENIX (43) and Coot (42, 44). Structure validation was performed with Molprobity (45). Model overfitting was evaluated through refinement against cryo-EM half maps (Fig. S1A to E). Figures were generated using UCSF Chimera and ChimeraX (41, 46).

### Data availability.

The cryo-EM data have been deposited in the Electron Microscopy Data Bank (EMDB) under accession codes 0243, 11900, 11901, 11902, and 11903 for the 50S and 70S, 0, 30, and 50 min incubation, respectively. The atomic models have been deposited in the Protein Data Bank (PDB) under IDs 6HMA, 7ASM, 7ASN, 7ASO, and 7ASP for 50S and 70S 0, 30, and 50 min incubation, respectively.
