# Supplementary material for: Structural Studies Reveal the Role of Helix 68 in the Elongation Step of Protein Biosynthesis
Source: mBio. 2022 Mar 29;13(2):e00306-22. doi: 10.1128/mbio.00306-22 (PMC9040758; doi:10.1128/mbio.00306-22)
Supplement: TABLE S1 [file mbio.00306-22-st001.pdf]

Supplementary Table 1: Cryo-EM data collection and model refinement (for all new structures)

Incubation	0 minutes	30 minutes		50 minutes	
Subunit	50S	50S	70S	50S	70S
**Data Collection**					
Microscope	Titan Krios				
Camera	Gatan K2	Falcon III	Gatan K2	Falcon III	Gatan K2
Voltage (kV)	300				
Magnification	29000	127000	29000	127000	29000
Pixel size ($Å.px^{-1}$)	0.858	1.1	0.858	1.1	0.858
Defocus range (μM)	(-0.7)-(-1.3)	-0.5 -(-1.50)	(-0.7)-(-1.3)	-0.5 -(-1.50)	(-0.7)-(-1.3)
Total dose ($e/Å^2$)	40				
Micrographs collected	2074	1671	2532	2079	3951
**Refinement**					
Number of particles (autopicked)	507852	291403	494694	457948	456794
Number of particles (used for 3D reconstruction)	211046	183484	144195	145354	164877
Resolution (Å; at FSC[a] = 0.143)	2.65	2.48	3.11	2.73	2.86
CC (model to map fit)	0.691	0.8795	0.62	0.4175	0.84
**Model Quality**					
Bonds (˚)	0.01	0.01	0.007	0.007	0.008

Angles (˚)	1.08	0.803	0.926	1.041	1.046
Chirality (˚)	0.055	0.05	0.081	0.079	0.066
Planarity (˚)	0.007	0.005	0.009	0.009	0.008
**Validation**					
Clashscore	4.62	5.35	26.47	13.47	26.08
**Proteins**					
MolProbity score	1.55	1.80	3.55	2.22	3.66
Rotamer outliers (%)	0.64	0.20	15.47	0.39	24.26
Ramachandran favored (%)	86.15	92.11	76.08	88.7	82.33
Ramachandran allowed (%)	13.32	7.69	22.05	10.91	14.84
Ramachandran outliers (%)	0.53	0.2	1.87	0.08	2.83
**RNA**					
Correct sugar pucker (%)	99.8	99.66	98.54	98.31	98.83
Correct backbone conformation	84.35	81.95	51.97	65.23	51.38
