# Supplementary figures and images for: Structural Studies Reveal the Role of Helix 68 in the Elongation Step of Protein Biosynthesis
Source: mBio. 2022 Mar 29;13(2):e00306-22. doi: 10.1128/mbio.00306-22 (PMC9040758; doi:10.1128/mbio.00306-22)

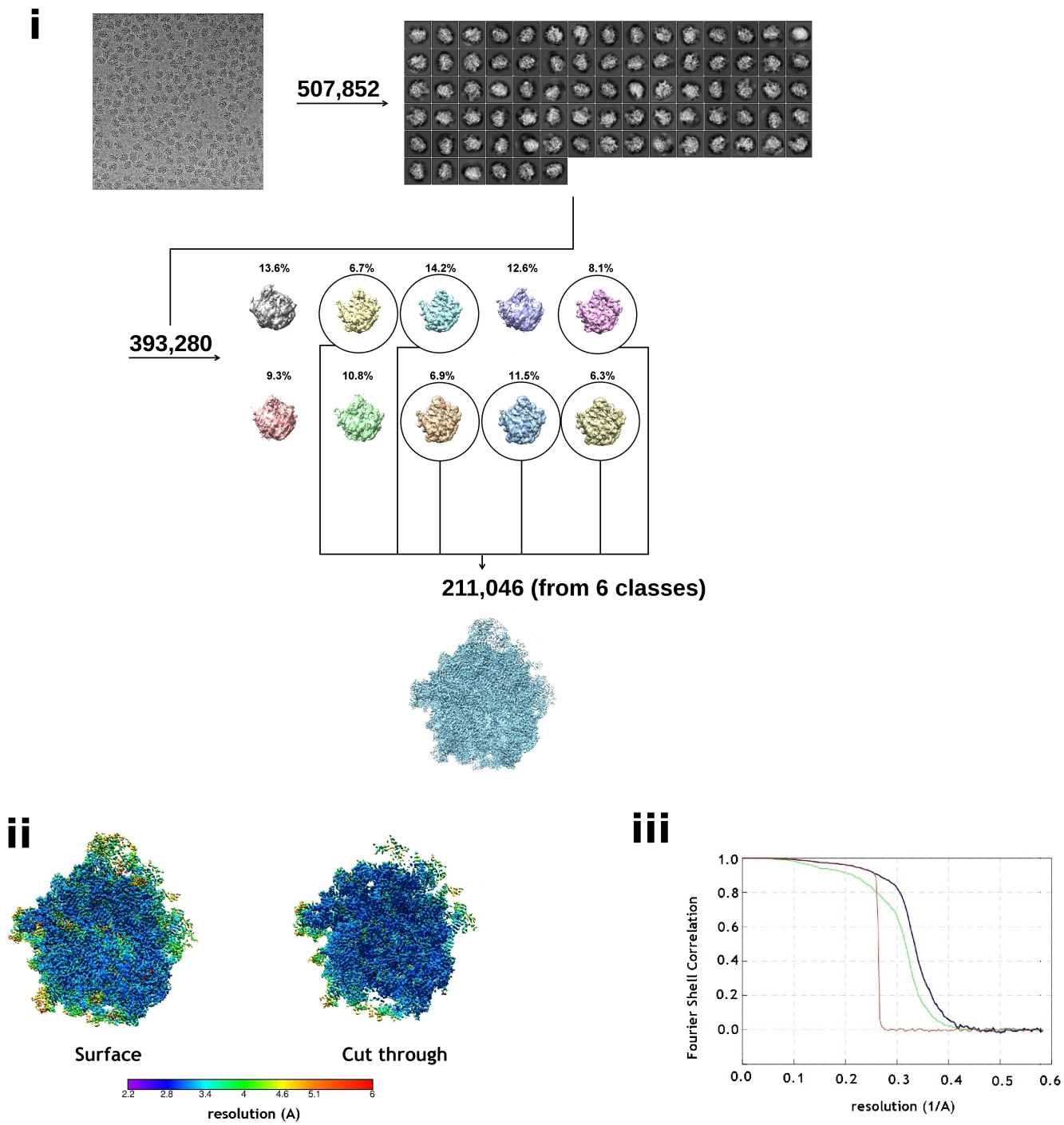

Fig. S1. Panel A

**i**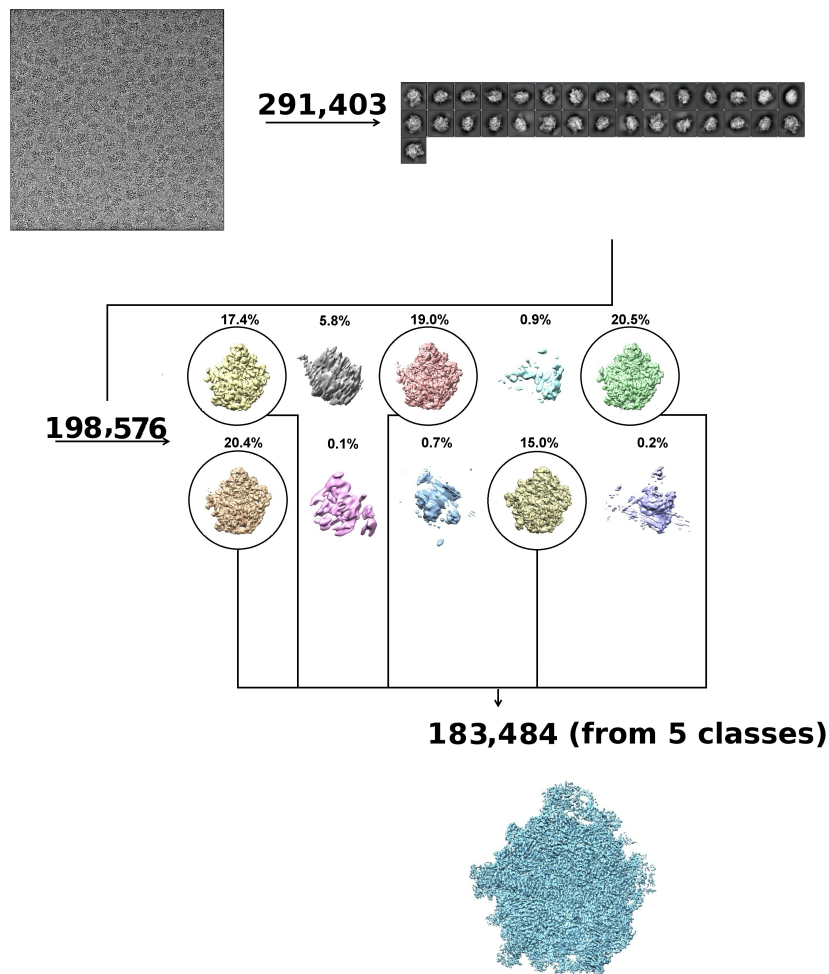**ii**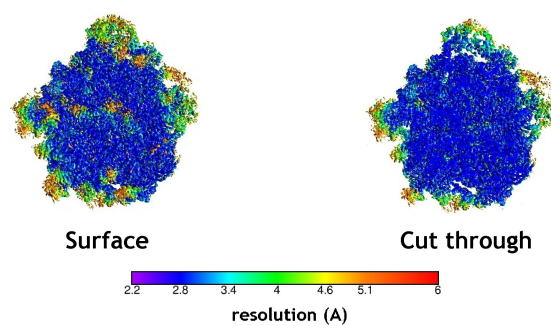**iii**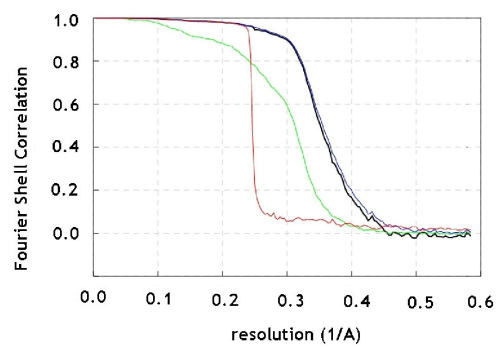

Fig. S1. Panel B

**i**

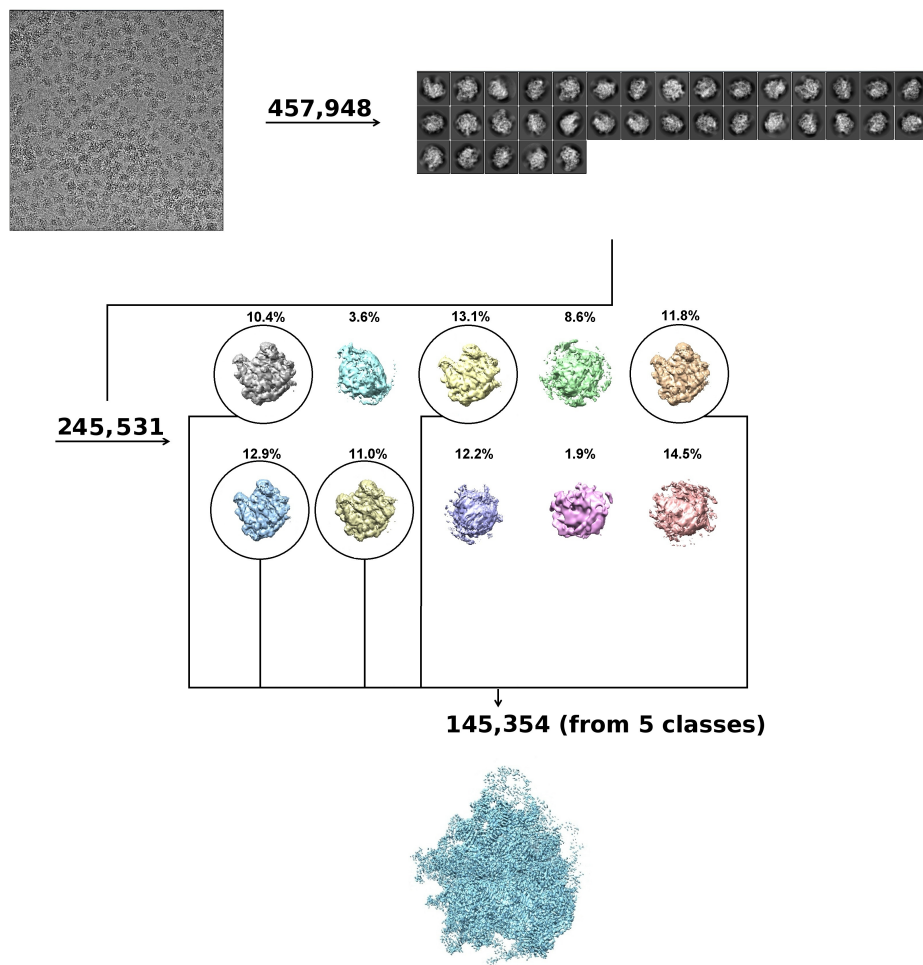

**ii**

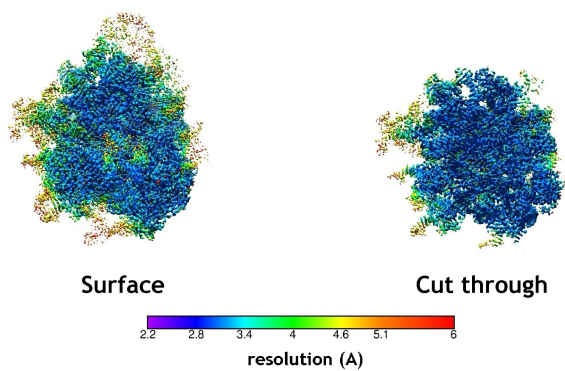

**iii**

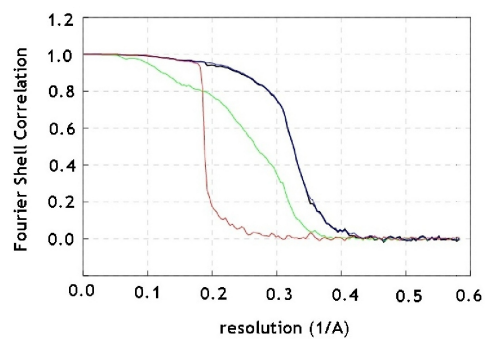

Fig. S1. Panel C

**i**

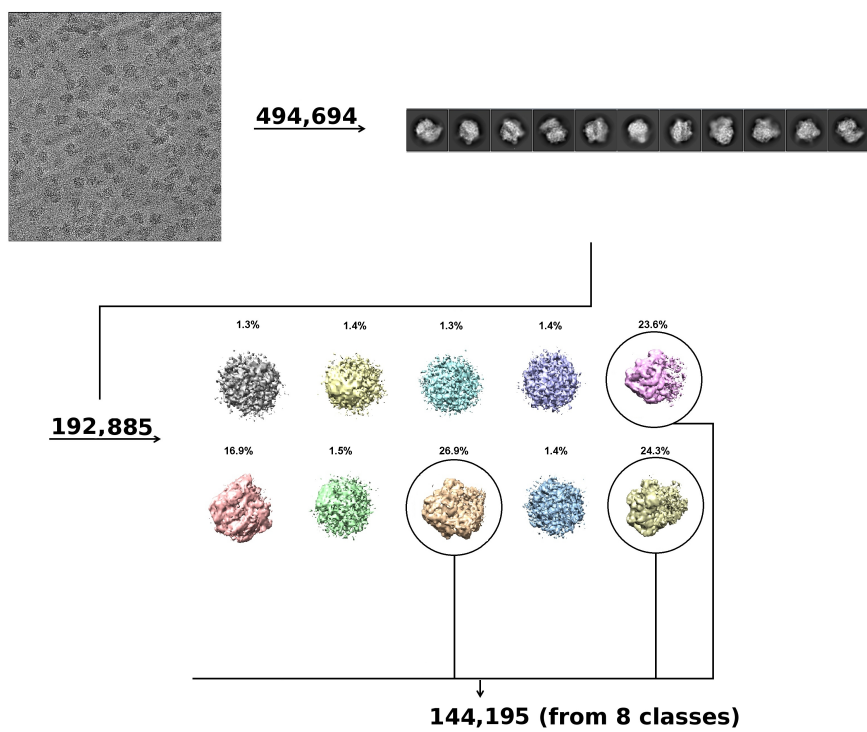

**ii**

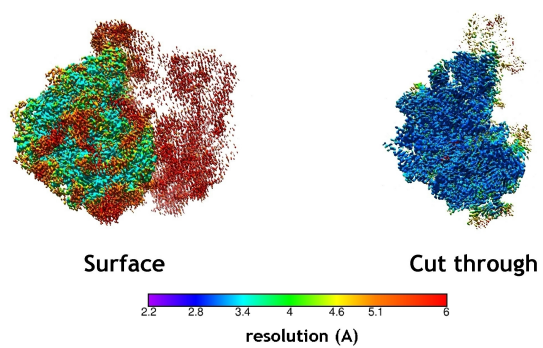

**iii**

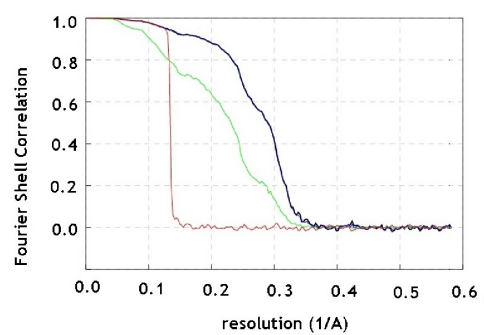

Fig. S1. Panel D

**i**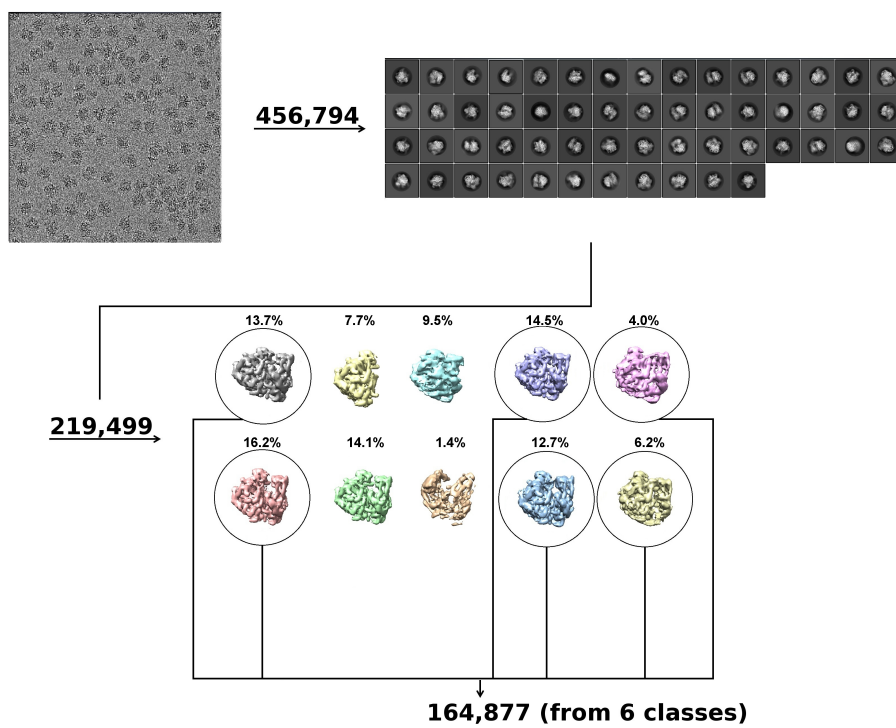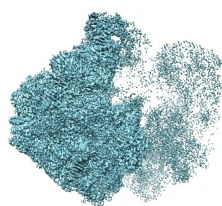**ii**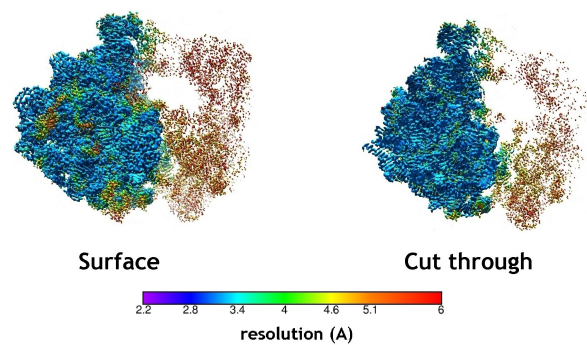**iii**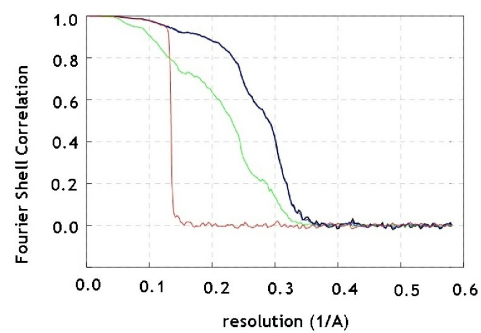

Fig. S1. Panel E

Supplement: FIG S1 [file mbio.00306-22-sf001.pdf]

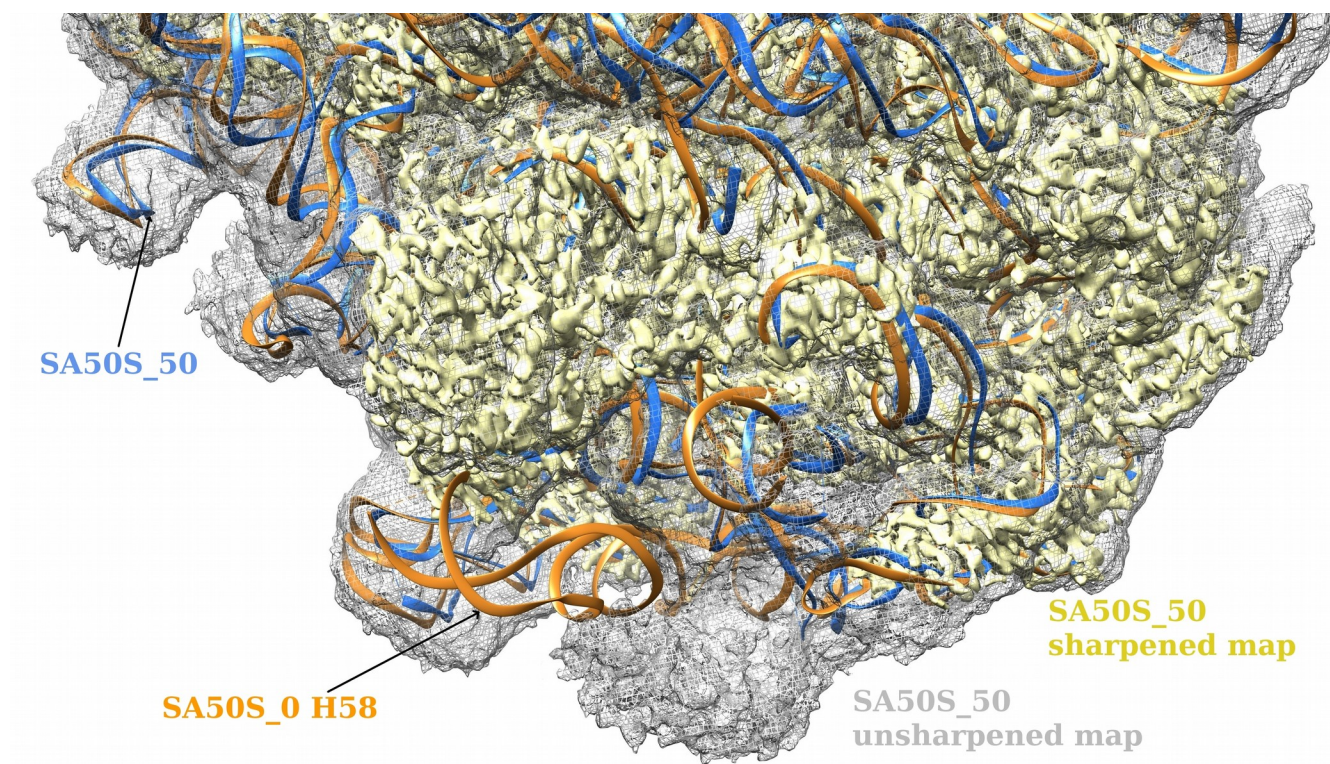

Figure S2

Supplement: FIG S2 [file mbio.00306-22-sf002.pdf]

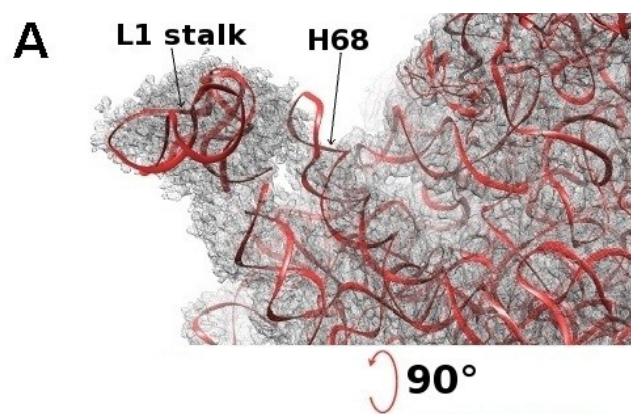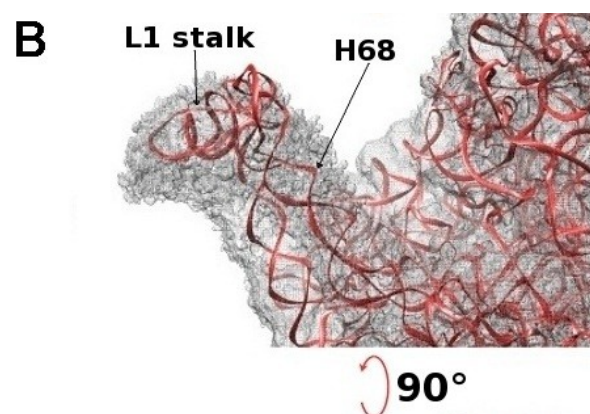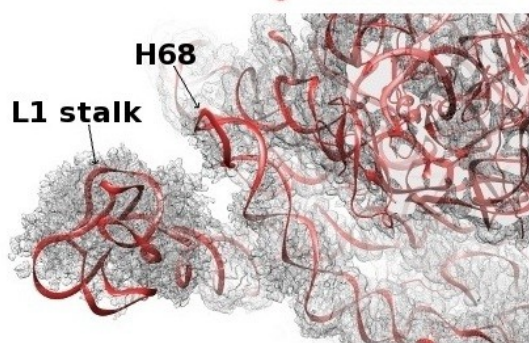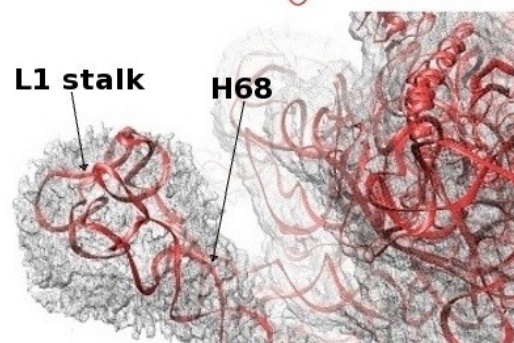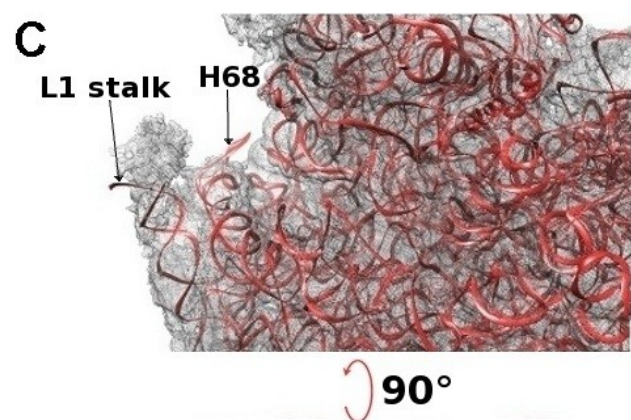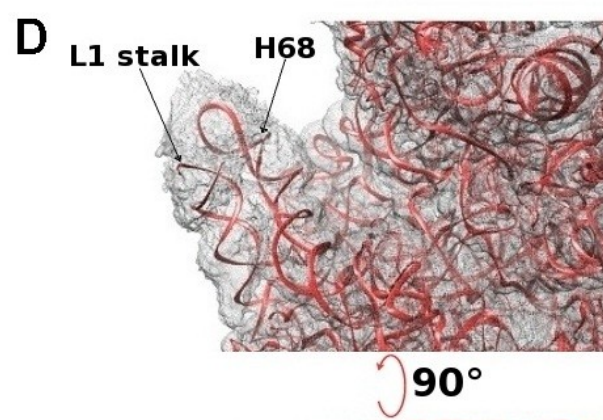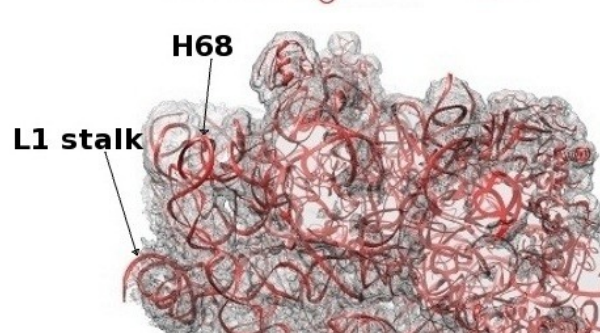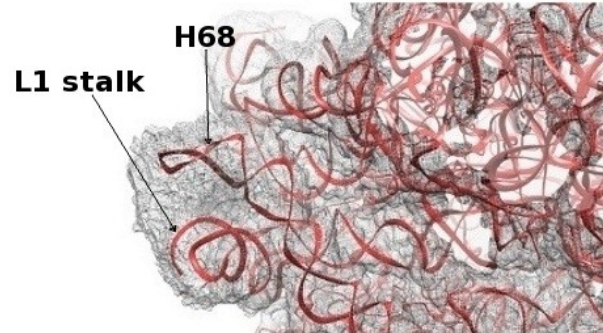

Figure S3

Supplement: FIG S3 [file mbio.00306-22-sf003.pdf]

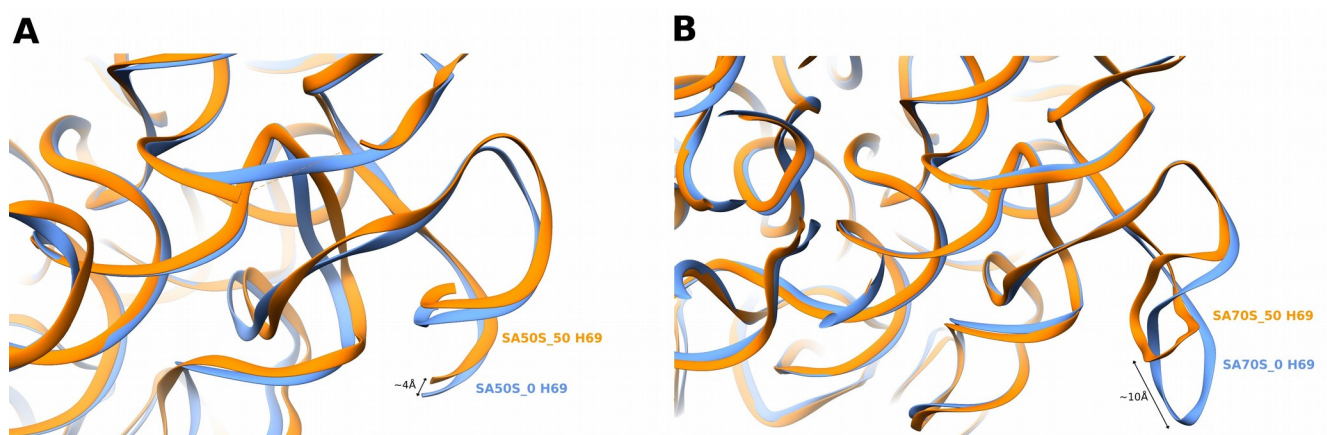

Figure S4

Supplement: FIG S4 [file mbio.00306-22-sf004.pdf]

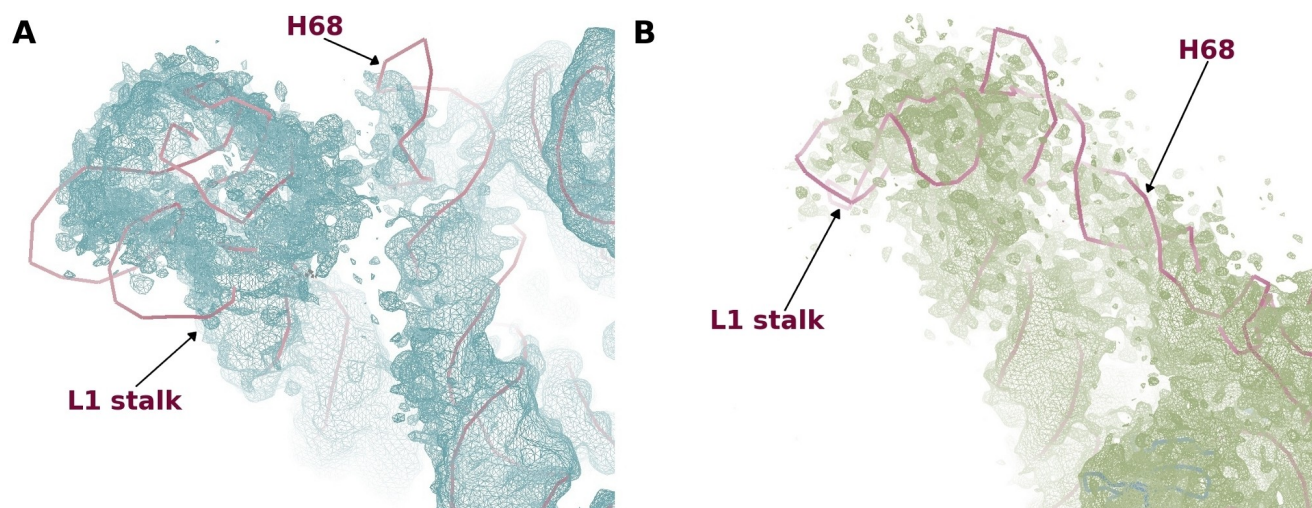

Figure S5

Supplement: FIG S5 [file mbio.00306-22-sf005.pdf]

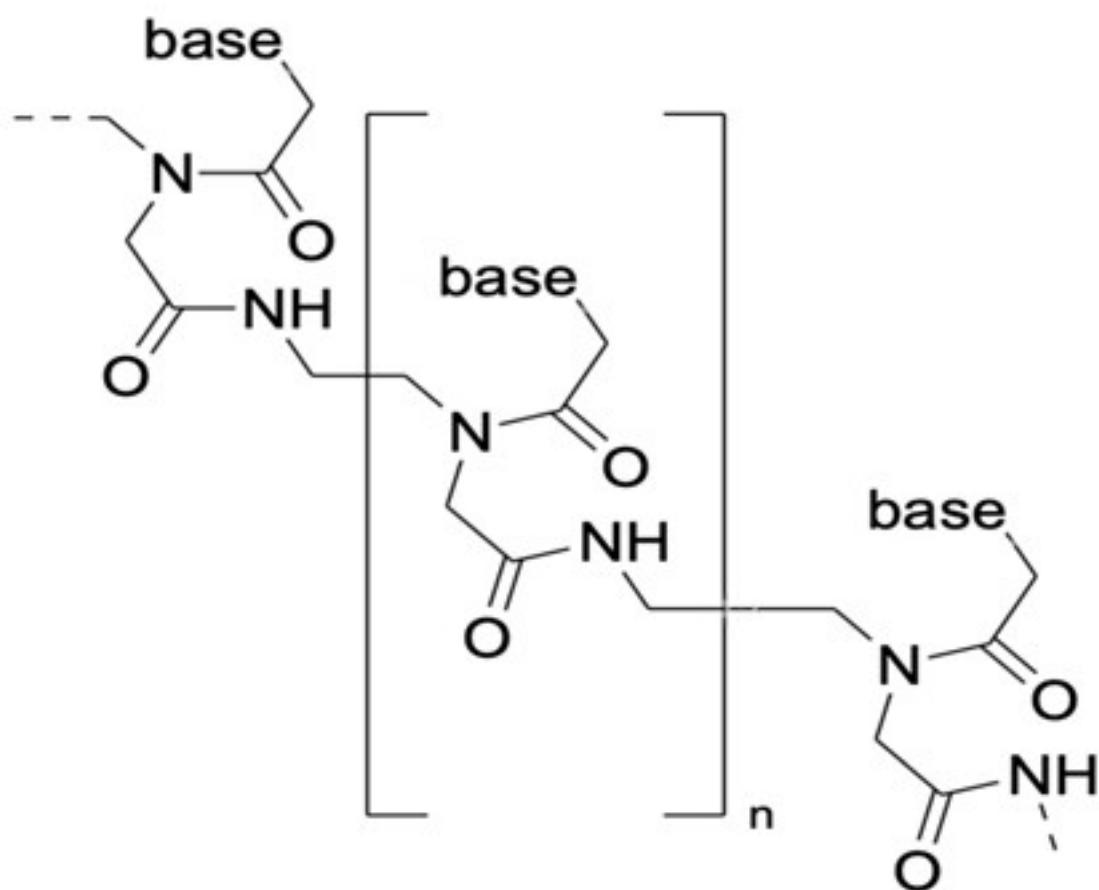

Figure S6

Supplement: FIG S6 [file mbio.00306-22-sf006.pdf]
