# Supplementary material for: Structural Studies Reveal the Role of Helix 68 in the Elongation Step of Protein Biosynthesis
Source: mBio. 2022 Mar 29;13(2):e00306-22. doi: 10.1128/mbio.00306-22 (PMC9040758; doi:10.1128/mbio.00306-22)
Supplement: FIG S7 [file mbio.00306-22-sf007.pdf]

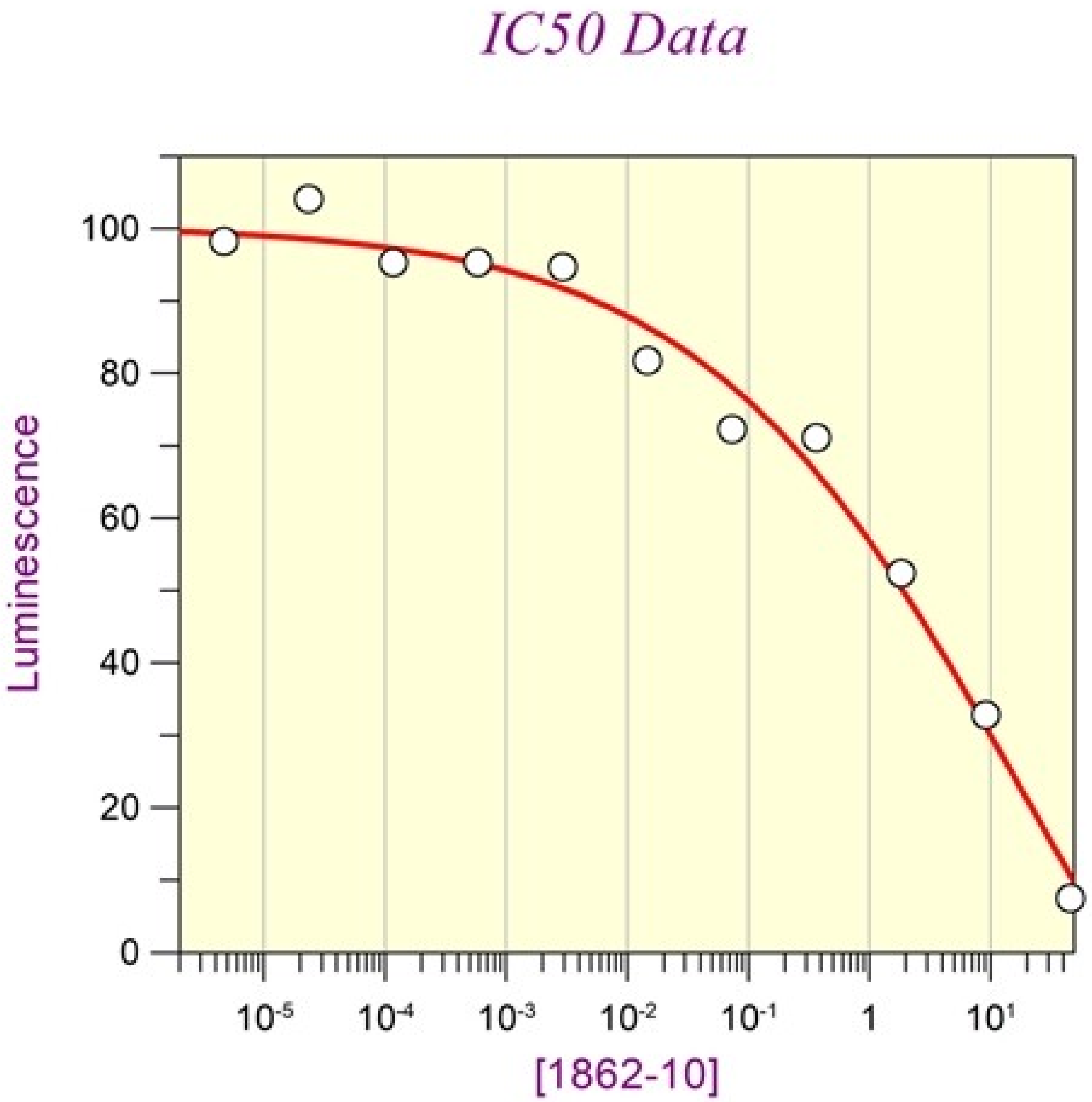

Parameter	Value	Std. Error
Y Range	159,3372	10,3268
IC 50	20,6114	8,6574
Slope factor	0,3221	0,0359

Figure S7A

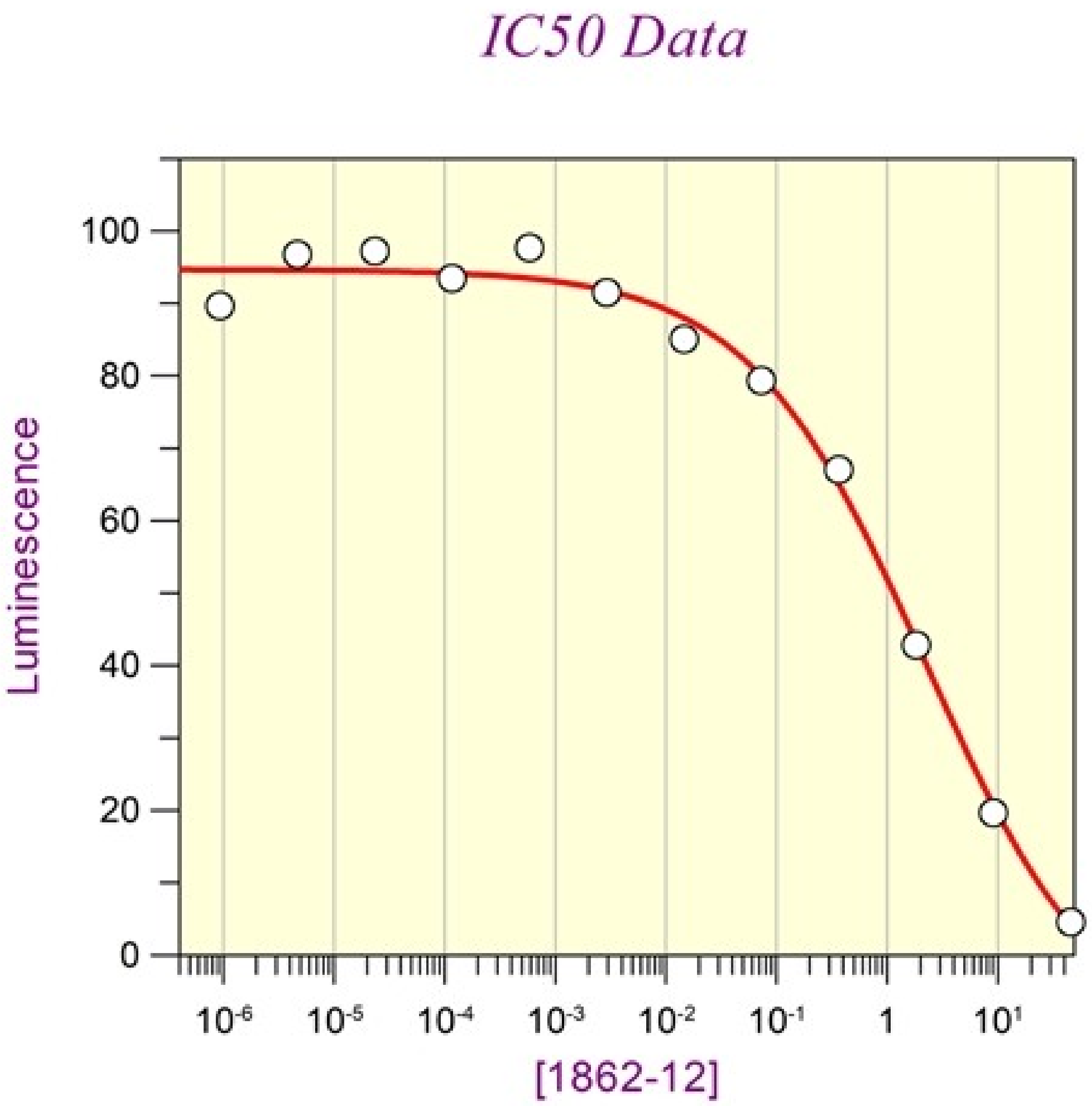

Parameter	Value	Std. Error
Y Range	108,1077	10,5881
IC 50	2,1905	0,9595
Slope factor	0,5425	0,0755

Figure S7B

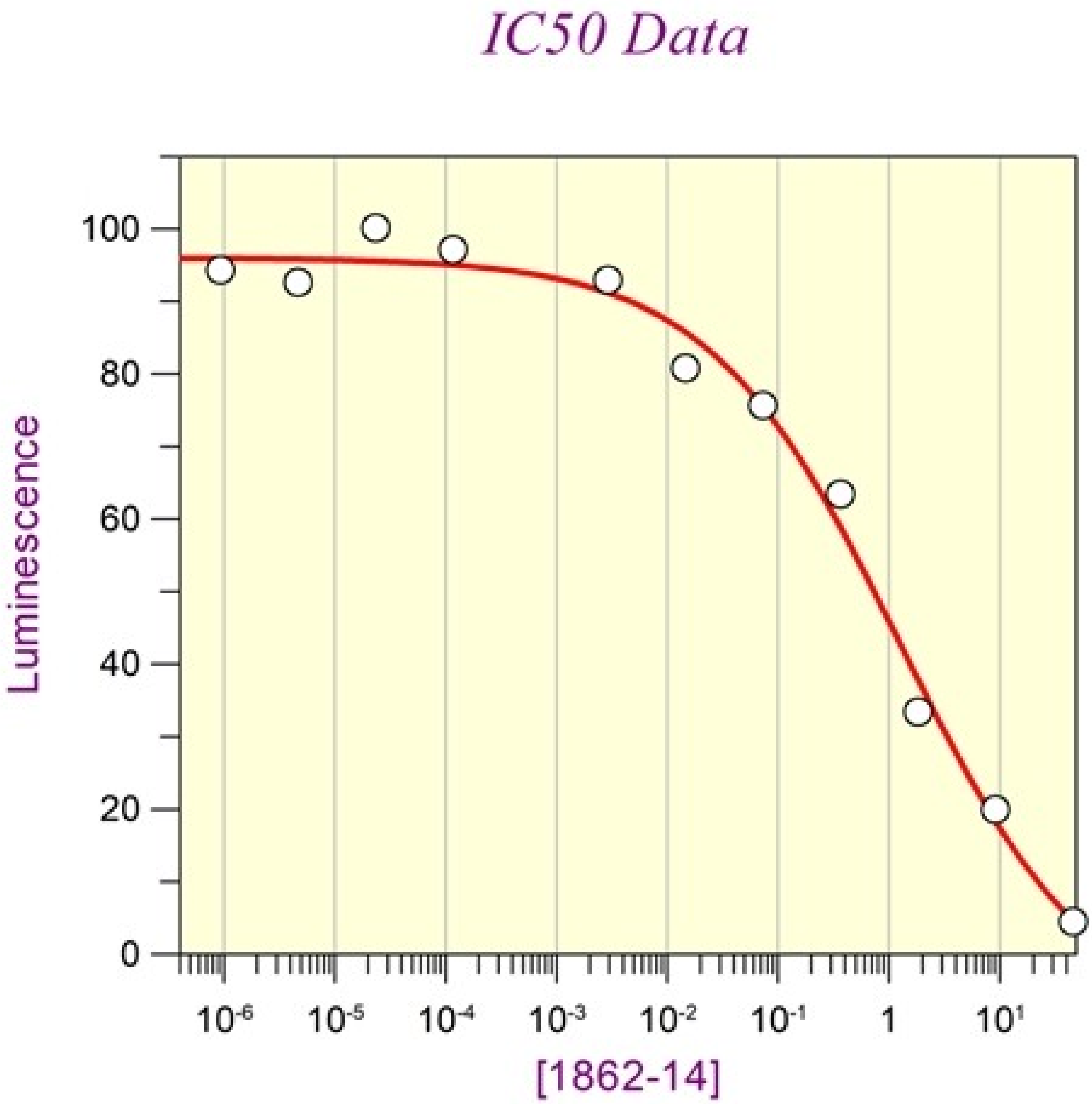

Parameter	Value	Std. Error
Y Range	106,5107	12,5714
IC 50	1,2640	0,7154
Slope factor	0,5017	0,0902

Figure S7C

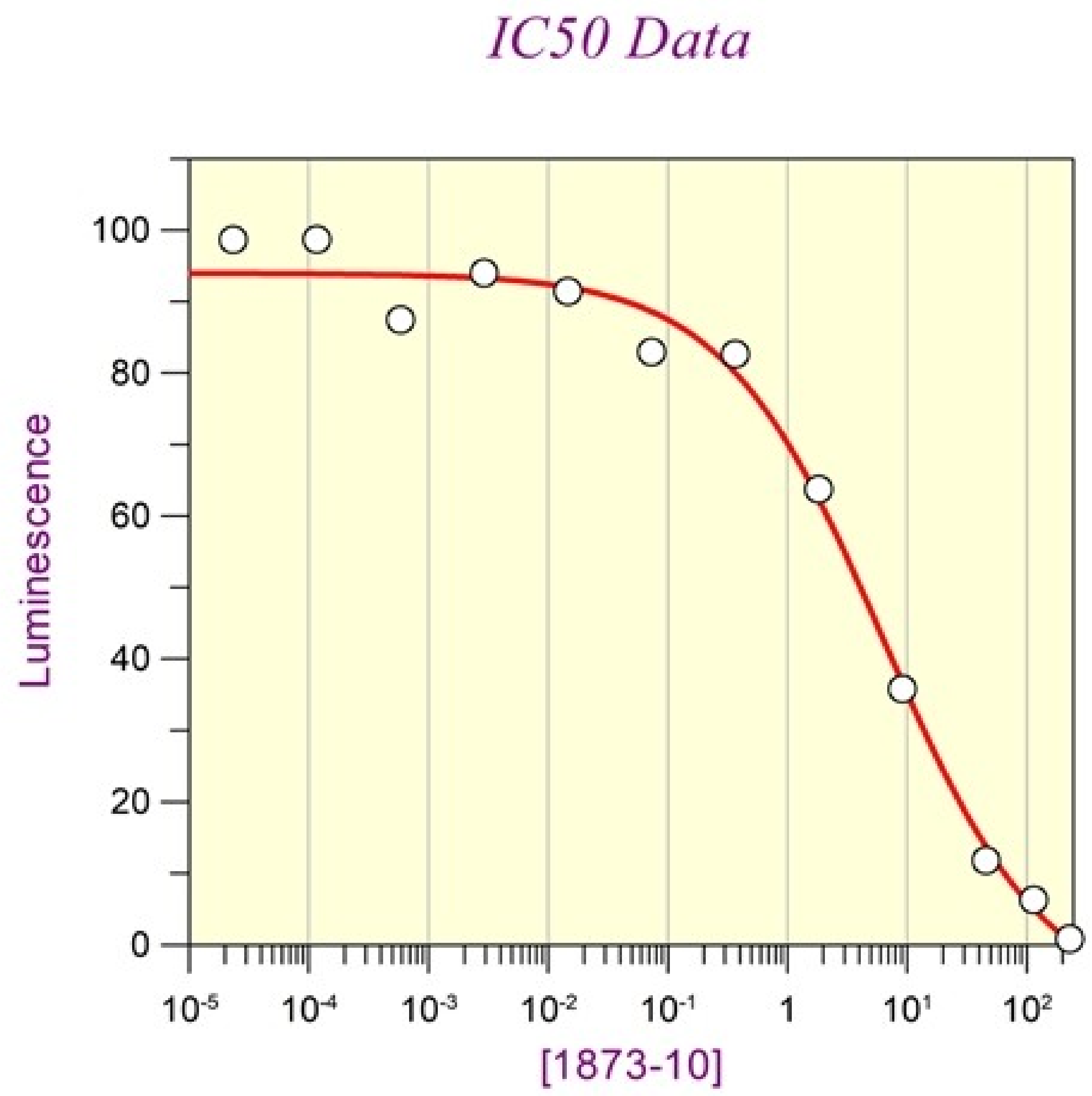

Parameter	Value	Std. Error
Y Range	102,5131	9,0726
IC 50	6,3506	2,3806
Slope factor	0,6493	0,1119

Figure S7D

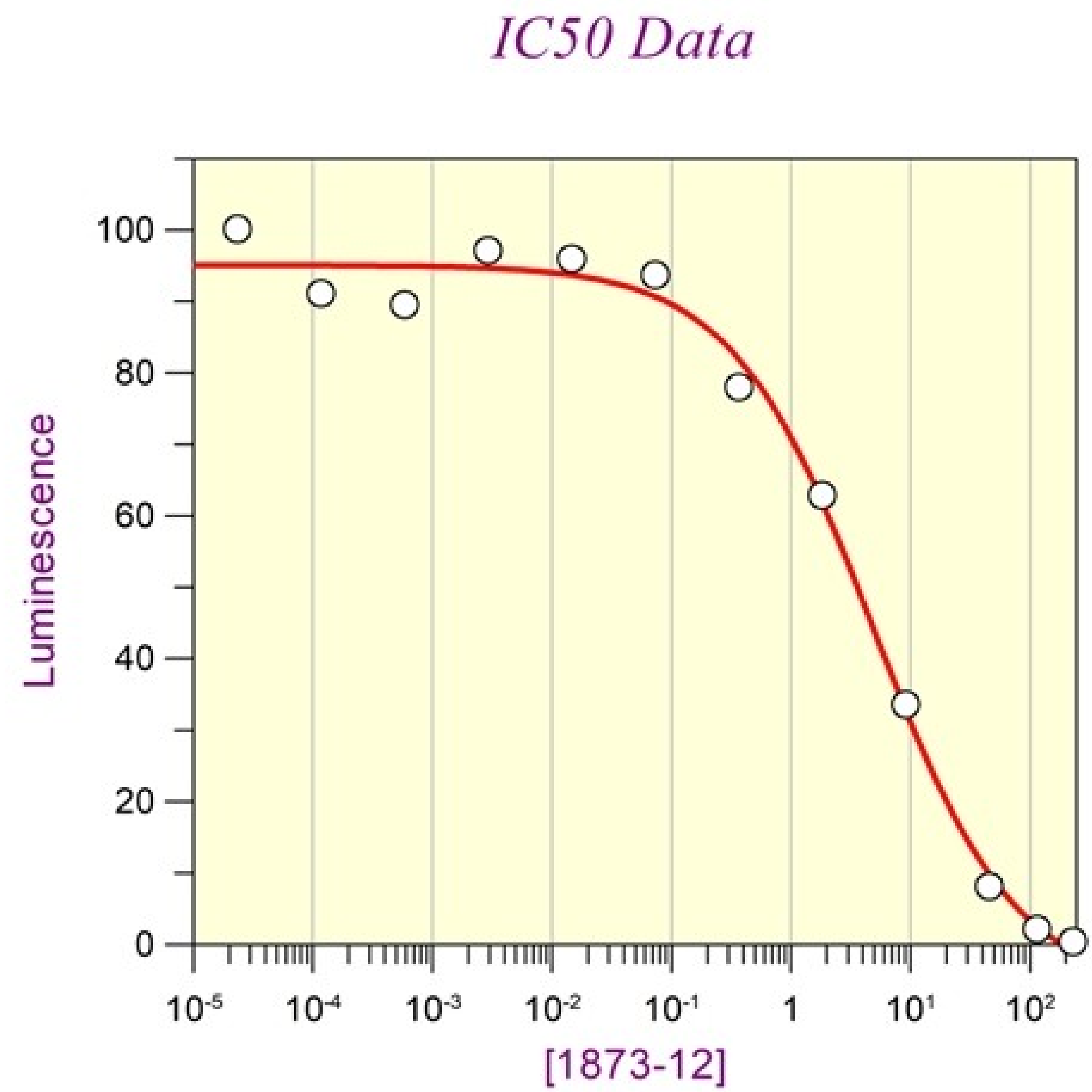

Parameter	Value	Std. Error
Y Range	101,6656	6,2575
IC 50	4,8944	1,2144
Slope factor	0,7359	0,1086

Figure S7E

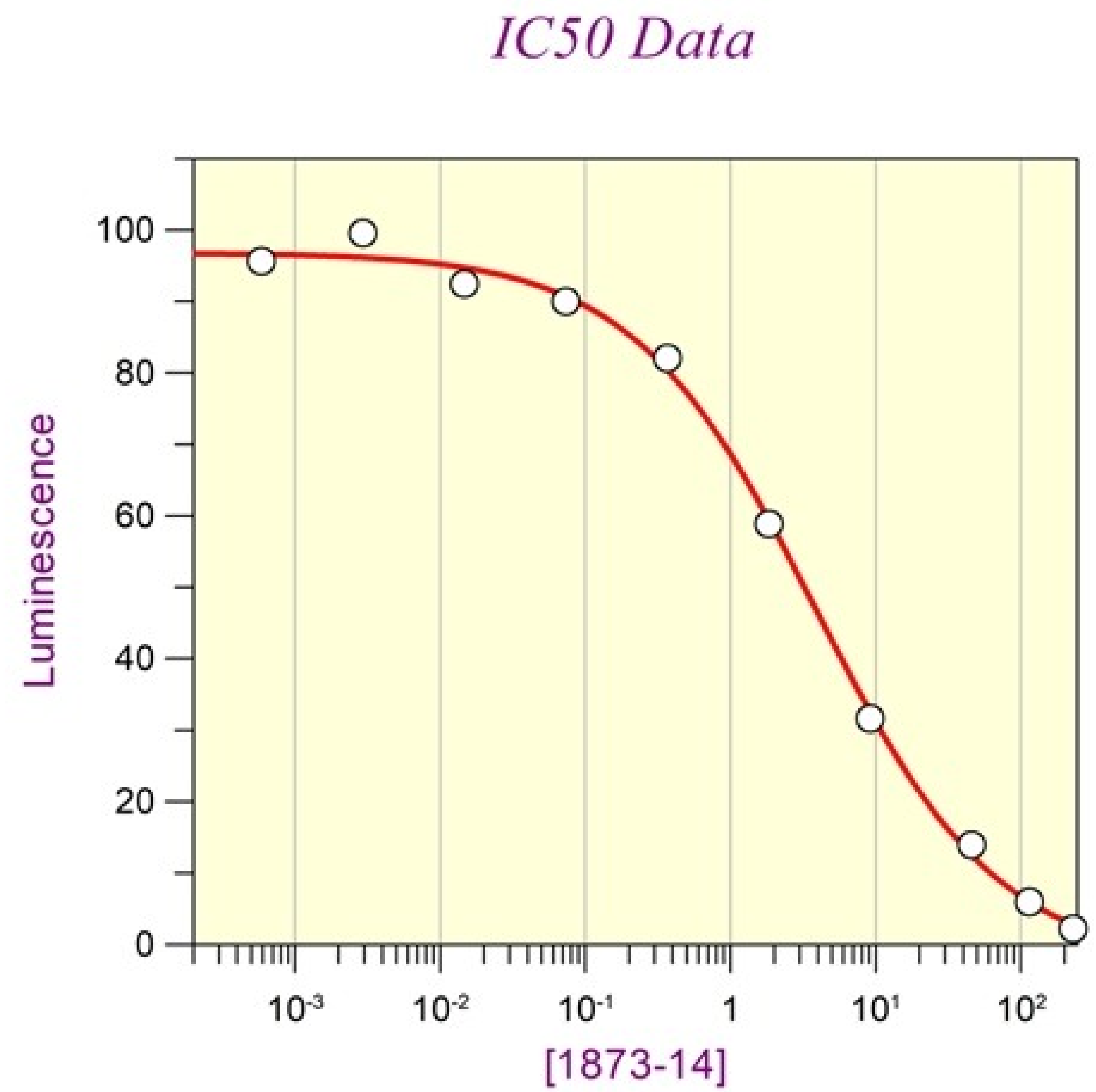

Parameter	Value	Std. Error
Y Range	99,6637	3,7326
IC 50	3,8544	0,5536
Slope factor	0,6888	0,0610

Figure S7F

i

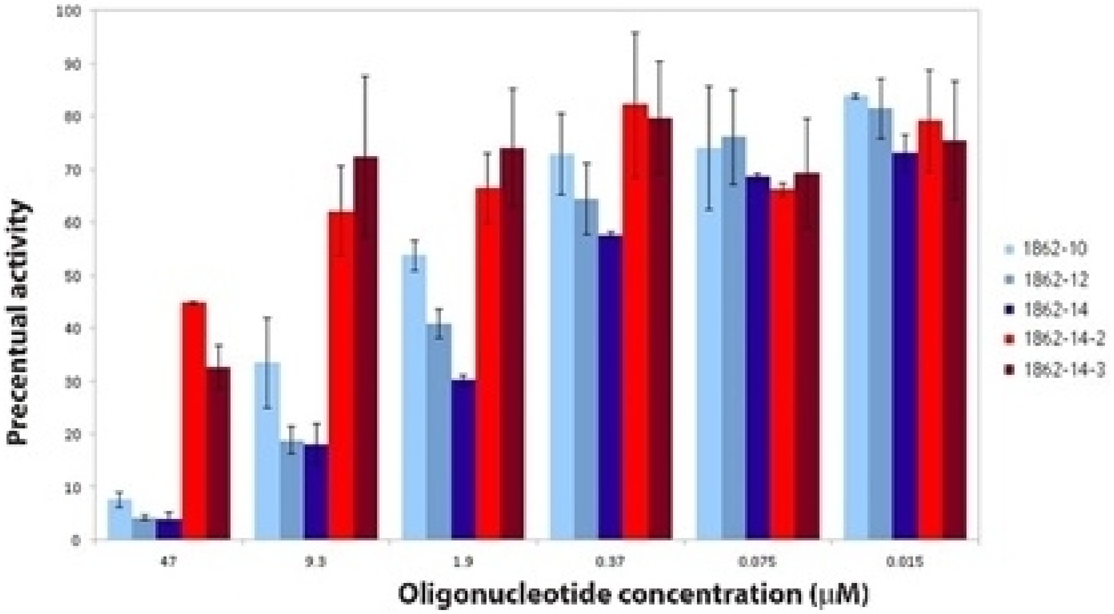

ii

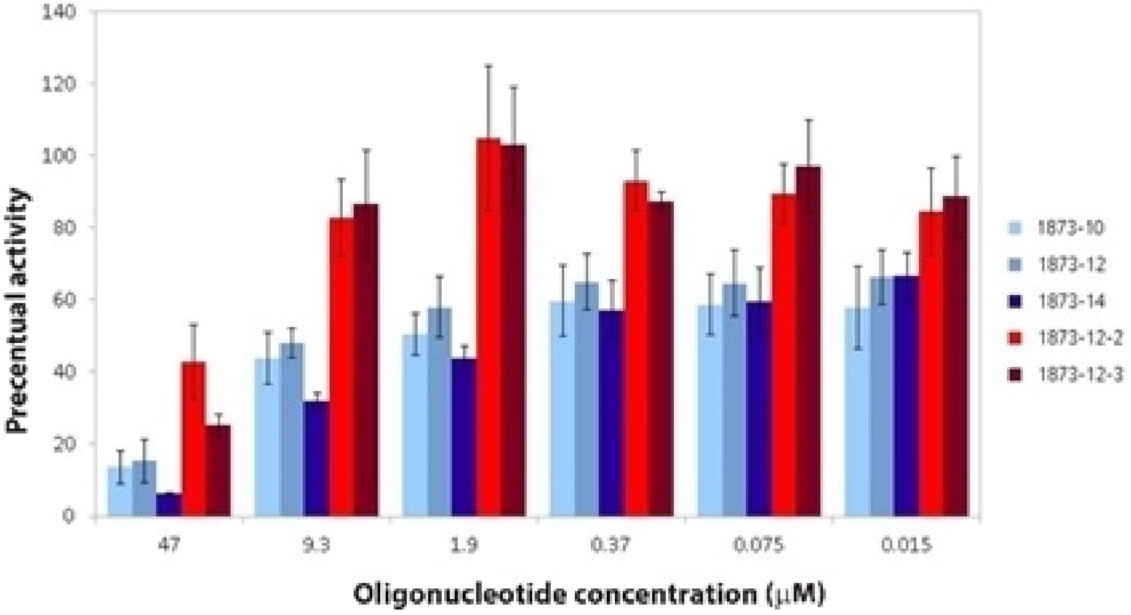

Figure S7. Panel G
